# Enhanced antibacterial performance of chili pepper seed protein hydrolysates by high hydrostatic pressure assisted enzymatic hydrolysis on *Staphylococcus aureus*: structural basis and mechanistic insights

**DOI:** 10.1016/j.fochx.2026.104296

**Published:** 2026-08-08

**Authors:** Sidi Liu, Yue Han, Yiyi He, Yunkai Zhang, Yu Xiong, Wenshu Huang, Dong Yang, Liang Zhao, Xiaojun Liao

**Affiliations:** a College of Food Science and Nutritional Engineering, China Agricultural University, National Engineering Research Center for Fruit & Vegetable Processing, Key Laboratory of Fruit & Vegetable Processing, Ministry of Agriculture and Rural Affairs, Beijing Key Laboratory for Food Non-thermal Processing, Beijing 100083, China; b College of Food Science and Pharmacy, Xinjiang Agricultural University, Urumqi 830052, China; c Faculty of Science, The University of Hong Kong, Pok Fu Lam Road, Hong Kong Special Administrative Region of China

**Keywords:** Chili pepper seed protein, By-product, Enzymatic hydrolysis, High hydrostatic pressure, Antibacterial activity

## Abstract

Antibiotic resistance of *Staphylococcus aureus* threatens public health. Plant by-product protein hydrolysates contain antibacterial peptides as antibiotic alternatives, but compact native protein structures restrict hydrolysis efficiency and antibacterial fragment release. This study applied high hydrostatic pressure (HHP) to assist papain hydrolysis of chili pepper seed protein (PSP) to improve hydrolysis efficiency and antibacterial activity. Pressurisation (100–500 MPa) enhanced PSP hydrolysis degree by 4.48–28.84%. Hydrolysis at 100 MPa gave rise to hydrolysate optimal antibacterial activity with 18.90% and 10.25% higher inhibition rates and zones. Structural and physicochemical analysis indicated that hydrolysis at 100 MPa increased the *α*-helix content by 23.15%, surface hydrophobicity by 20.50%, < 3 kDa peptides by 284.07%, and improved solubility and dispersion. These variations strengthen hydrolysate capacity on membrane binding and disruption, and form a favorable antibacterial microenvironment. These results provide an effective strategy to optimize enzymatic hydrolysis and high-value utilization of chili pepper seeds.

## Highlights

• HHP pretreatment unfolded PSP and improved enzymic hydrolysis efficiency.
• The changes in antibacterial activity of PSPE under different HHP conditions has been revealed.
• HHP-induced structural and physicochemical changes relate to enhance the bioactivity of PSPE against *S. aureus*.
• Optimal HHP (100 MPa) enhanced bioactivity against *S. aureus* by 18.90% compared to control.
• This study provides new insights for the high-value utilization of chili pepper seeds.

## 1 Introduction

The accelerated evolution of bacterial antimicrobial resistance constitutes a critical threat to global public health, food safety, and ecological security. Among resistant pathogenic bacteria, *Staphylococcus aureus* (*S. aureus*) represents a predominant public health challenge in the 21st century, causing higher global mortality than other resistant bacteria (Liu et al., 2021). In the United States, *S. aureus* bloodstream infections result in approximately 20,000 annual deaths (Piewngam et al., 2023). This crisis is primarily attributed to the fact that the efficacy of conventional single-target antibiotics is progressively compromised by microbial adaptive mutations, driving demand for novel antimicrobial alternatives (Darby et al., 2024; Zhang et al., 2021).

Distinct from conventional antibiotics, antibacterial peptides exhibit multi-modal membrane-targeting modes of action derived from their structural diversity, which greatly impede the evolution of antibiotic-resistant mutants while exerting broad-spectrum activity and low cytotoxicity (Huan et al., 2020; Mba & Nweze, 2022; Sneideris et al., 2023). Therefore, continuous exploration and discovery of novel antibacterial peptides from diverse biological sources have become a global imperative mission to combat antimicrobial resistance (Sun, 2024). Enzymatic hydrolysis of plant proteins releases abundant latent “encrypted” antibacterial peptides, which accumulate in hydrolysates and serve as a novel resource reservoir for natural antibacterial peptide excavation (Chen, Song, et al., 2025). This approach is characterized by material sustainability, mild processing conditions, and cost-effectiveness relative to chemical synthesis and microbial fermentation (Ngoh & Gan, 2016; Rivero-Pino et al., 2023). Numerous studies have reported the preparation of antibacterial hydrolysates from various plant proteins, including *Moringa oleifera* seed protein (Zhao et al., 2022), pea protein (Wang et al., 2025), seabuckthorn seed protein (Zheng et al., 2024), and Chia Seed (*Salvia hispanica* L.) protein (Aguilar-Toalá et al., 2020). However, the compact folded conformation, low solubility, molecular aggregation, and heterogeneous dispersion of native plant proteins impede enzyme accessibility to buried cleavage sites, resulting in a low degree of hydrolysis and restricting the release of “encrypted” peptide fragments (Dent et al., 2024). Above constraints constitute the core bottleneck restricting the bioactivity of plant protein hydrolysate.

Non-thermal pretreatments, including ultrasound (Pacheco et al., 2025), electron beam irradiation (Zhang et al., 2023) and high hydrostatic pressure (Liu, Hou, et al., 2025), have been demonstrated to unfold protein conformation, improve protein physicochemical properties, and hydrolysis efficiency without inducing thermal degradation. High hydrostatic pressure (HHP) as a non-thermal processing technique, could modulate secondary and tertiary structures of plant proteins at ambient temperature while preserving amino acid sequences, enzyme specificity, and peptide functionality (Ana et al., 2021; Queirós et al., 2018). Existing studies of HHP-assisted enzymatic hydrolysis mainly focus on plant protein hydrolysates with antioxidant (Zhang, Jing, et al., 2022) and angiotensin-converting enzyme (ACE) - inhibitory (Nazir et al., 2020) activities. Unfortunately, the preparation of antibacterial hydrolysates via HHP assisted enzymatic hydrolysis has received limited attention in existing literature, and the underlying mechanisms of its effect on antibacterial activity remain unclear. Insufficient systematic research on HHP parameters restricts precise optimization of hydrolysis degree and antibacterial activity of plant protein hydrolysates and hinders subsequent research and industrial-scale production. Accordingly, it is necessary to systematically explore the pressure induced structure-activity relationship of plant protein hydrolysates and reveal the molecular mechanism by which HHP influences the change in antibacterial activity.

China is the leading global producer of chili pepper, with an annual yield of 28 million tons. Chili pepper seeds constitute 30–50% (dry weight) of chili peppers and contain 17–19.83% protein on a dry basis (Deng et al., 2025). The chili pepper seed protein (PSP) exhibited elevated concentrations of essential amino acids, ranging from 2.77% to 4.44%, and total aromatic amino acids relative to the FAO/WHO reference pattern (Liu, Pan, et al., 2025; Wang, Ma, et al., 2021). Currently, most chili pepper seeds are discarded as agricultural waste or processed into low-grade animal feed, which results in substantial waste of protein resources and potential environmental pollution (Wang, Wang, et al., 2021). Therefore, the production of antibacterial hydrolysates via extraction of chili pepper seed protein (PSP) benefits from sufficient raw materials and low manufacturing expenses, which offers a high-value utilization pathway for chili pepper processing by-products.

Our previous work has established a PSP extraction protocol and found that HHP treatment was observed to induce conformational unfolding and enhance the functional properties of PSP (Liu, Pan, et al., 2025). Furthermore, we have successfully obtained antibacterial hydrolysates of PSP (PSPE) via papain hydrolysis. Those previous results provide solid material, technical and theoretical foundations for further research on HHP - assisted enzymatic hydrolysis of PSP (HHP-PSPE). However, the effects of HHP on the antibacterial activity of PSP, along with the underlying molecular mechanisms, remain unclear.

Based on the above, this work innovatively applies HHP in assisting enzymatic hydrolysis to prepare antibacterial PSPE. The objective of this work was to explore the impact of HHP under 100–500 MPa on degree of hydrolysis (DH), antibacterial activity, structure and physicochemical properties of PSPE, elucidate relevant underlying mechanisms, and characterize structure – antibacterial activity relationship. These findings will fill the research gaps in HHP-assisted enzymatic hydrolysis for plant-derived antibacterial protein hydrolysates, establish fundamental insights into the structure – bacteriostatic activity correlation of plant protein hydrolysates, provide a new resource reservoir and material to prepare new antimicrobial peptides, and offer a green and efficient method for the high-value utilization of chili pepper seeds.

## 2 Method and materials

### 2.1 Materials and sample preparation

#### 2.1.1 Materials

Honglong 23 chili pepper seeds were obtained from Xinjiang Tianjiao Hongan Agriculture Technology Co., Ltd. (Xinjiang Province, China). All reagents and chemicals were obtained from Beijing Solarbio Science & Technology Co., Ltd. (China) and Sinopharm Chemical Reagent Co., Ltd. (China). *S. aureus* (ATCC 25923) was employed as the model Gram-positive bacterium and obtained from the China Industrial Microbial Culture Collection Center.

#### 2.1.2 Extraction of PSP

PSP was isolated by alkali solution and acid sedimentation as described previously with minor modifications (Liu, Pan, et al., 2025). Dried chili seeds were fully pulverized with a grinder (XL-600B, Yongkang Xiaobao Electric Appliance Co., Ltd., China) and passed through a 100-mesh sieve for subsequent use. A quantity of 500.0 g of powder was mixed with 10.0 L of distilled water. The suspension was adjusted to pH 10.0 with 0.5 mol/L NaOH, stirred continuously for 5 h with a stirrer (SHJ-6A, Jiangsu Dongpeng Instrument Manufacturing Co., Ltd., China) at room temperature, and then stood at 4 °C for 4 h in a refrigerator (GR-A2078DSF, LG Electronics Inc., South Korea). After centrifugation at 10,600×*g* for 20 min using a high-speed refrigerated centrifuge (CF-RXII, Hitachi Ltd., Japan‌), the supernatant was collected and adjusted to pH 4.5 using 0.1 mol/L HCl, followed by stirring for 4 h at room temperature and standing at 4 °C for 4 h in a refrigerator. The precipitate was har*v*ested by centrifugation at 10,600×*g* for 20 min using a high-speed refrigerated centrifuge (CF-RXII, Hitachi Ltd., Japan‌). The obtained precipitate was redissolved in deionized water (1:10, w/v), and the solution pH was adjusted to 7.5. Subsequently, the sample solution was placed in a dialysis bag and dialyzed against deionized water for 24 h. The dialyzed solution was poured into 16.0 mm-diameter glass petri dishes, pre-frozen at −80 °C in a refrigerator (DW-86L578J, Qingdao Haier Biomedical Co., Ltd., China) for 24 h, and then lyophilized for 48 h using a freeze dryer (LSCplus, Christ, Germany). The final powder was sie*v*ed through a 100-mesh screen, sealed, and stored at 4 °C in a refrigerator (GR-A2078DSF, LG Electronics Inc., South Korea) for subsequent experiments.

#### 2.1.3 HHP assisted enzymatic hydrolysis

1.0 g of PSP was dispersed in 3.5 mL PBS solution (pH 7.5) at a solid-to-liquid ratio of 1:7 (w/v) in a 10.0 mL centrifuge tube, followed by continuous stirring for 24 h. The solution was transferred into polypropylene vacuum bags and hermetically sealed using a vacuum packaging machine (UB268, Deli Group Co., Ltd.)., China). The sealed samples were homogenized and treated with HHP (SHHP-5L, Shanxi Lidefu Technology Co., Ltd., China) at different pressures (0.1, 100, 200, 300, 400, 500 MPa) for 5 min under ambient temperature. After HHP pretreatment, the treated sample solution was transferred into 10.0 mL centrifuge tubes and adjusted to pH 7.5. Papain (800.0 U/mg) was then added to the solution to achieve a final enzyme activity of 6000.0 U/mL, and the mixture was continuously incubated with stirring at 50 °C for 4 h with a thermostatic water bath magnetic stirrer (SHJ-6A, Jiangsu Dongpeng Instrument Manufacturing Co., Ltd., China). During hydrolysis, the pH was readjusted to 7.5 every 0.5 h. Upon hydrolysis completion, the reaction solution was immediately heated in a 95 °C water bath for 10 min to terminate the enzymatic reaction (Bandyopadhyay & Ghosh, 2002; Panda et al., 2015). After cooling to ambient temperature, the mixture was centrifuged at 9739×*g* for 20 min with a centrifuge (MGL-16MA, Meiruike Instrument (Shanghai) Co., Ltd., China). The supernatant was collected, adjusted to neutral pH, freeze-dried, and stored at −20 °C refrigerator until further analysis. The resulting samples were denoted as PSPE, PSP-100E, PSP-200E, PSP-300E, PSP-400E, and PSP-500E, which indicate that the PSP were treated by HHP-assisted enzymatic hydrolysis at 0.1 MPa, 100 MPa, 200 MPa, 300 MPa, 400 MPa and 500 MPa. All HHP-treated samples are collectively referred to as HHP-PSPE.

### 2.2 Degree of hydrolysis (DH)

The degree of hydrolysis (DH), defined as the percentage of cleaved peptide bonds relative to the total number of peptide bonds in PSP, was determined by formaldehyde titration based on a previously study with minor modifications (Alahmad et al., 2022). Briefly, 0.2 g of PSPE and HHP-PSPE powder was separately dissolved in 5.0 mL of purified water under magnetic stirring using a magnetic stirrer (C-MAG HS 7, IKA Group, Germany) until complete dissolution. The pH of the solution was adjusted to 7.0 using 0.01 mol/L standard NaOH solution. Subsequently, 10.0 mL of a 0.5 mol/L formaldehyde solution was added, and the mixture was continuously stirred for 8 min at room temperature. Titration was performed using a standard 0.01 mol/L NaOH solution under agitation until a terminal pH of 8.5 was attained, with the consumed NaOH volume recorded as *V*_1_ (mL). A blank control, consisting of 5.0 mL of PSP solution at an equivalent concentration, was processed concurrently, following identical procedural parameters, recording the consumed NaOH volume denoted as V_0_ (mL). The DH value was calculated using Eq. (1):

(1) $$\mathrm{DH}\left(\%\right)=C\times \left(V_{1}-V_{0}\right)\times 0.014/N\times 100\%$$

(C: Concentration of the NaOH standard solution, (mol/L); V_1_: volume of NaOH standard solution consumed by the enzymatic hydrolysate, (mL); V_0_: volume of NaOH standard solution consumed by the blank solution, (mL); 0.014: the nitrogen milligram equivalent; N: Total nitrogen content of the sample, (g).)

### 2.3 Antibacterial properties

#### 2.3.1 Inhibition rate

The antibacterial activity of PSPE and HHP-PSPE was evaluated and compared in terms of bacterial inhibition rate. The sample concentration for antibacterial testing was fixed at 10 mg/mL. Specifically, 20.0 mg of PSPE and HHP-PSPE powder were accurately weighed and fully dissolved in 1.0 mL of sterile PBS solution (pH 7) to prepare the antibacterial solution. Overnight-grown cultures of *S. aureus* (ATCC 6538) grown in Luria-Bertani (LB) broth at 37 °C were collected and diluted with fresh LB to an optical density at 600.0 nm (OD_600_) of 0.8, yeast extract to attain a final concentration of approximately 1.0 × 10^6^ CFU/mL. Equal volumes (100.0 μL) of the bacterial suspension were incubated with serially-diluted peptide solutions in a 96-well microtiter plate. After incubation for 12 h at 37 °C in an electric constant-temperature incubator (DNP-9272, Shanghai Jinghong Experimental Equipment Co., Ltd., China), absorbance at 600 nm was determined to evaluate bacterial growth inhibition using a microtiter plate reader (Spark™ 10M, Tecan Group Ltd., Switzerland), with sterile PBS solution (pH 7) as the blank control. The bacterial inhibition rate was calculated according to the following formula:

(2) $$\text{Inhibition rate}\;\left(\%\right)=\left(A_{0}-A_{1}\right)/A_{0}\times 100\%$$

(A₀ and A₁: The OD₆₀₀ values of the control group and the sample, respectively.)

#### 2.3.2 Inhibition zone

The antibacterial activity of the samples against *S. aureus* was determined using the pour plate method with minor modifications (Sun et al., 2016). 10.0 mg of PSPE and HHP-PSPE powder was accurately weighed and fully dissolved in 1 mL of a sterile PBS solution (pH 7) to prepare the antibacterial solution (10.0 mg/mL). Overnight-grown cultures of *S. aureus* (ATCC 6538) grown in Luria-Bertani (LB) broth at 37 °C were collected and diluted with fresh LB to an optical density at 600 nm (OD_600_) of 0.8, and yeast extract to attain a final concentration of approximately 1 × 10^5^ CFU/mL. A 3.0 mL aliquot of the bacterial suspension was added to 300.0 mL of sterile melted LB agar, yielding a final bacterial concentration of approximately 1 × 10^3^ CFU/mL. The mixed melted LB agar was poured into 90.0 mm sterile petri dishes. After hardening, seven 6.0 mm diameter wells were made using a sterile borer. Subsequently, 150.0 μL of each antibacterial solution and sterile PBS solution (pH 7) were added into the wells. The plates were stored at 4 °C in a refrigerator (GR-A2078DSF, LG Electronics Inc., South Korea) for 1 h to facilitate sufficient diffusion of the samples into the agar, followed by incubation at 37 °C for 12 h. The diameter of the inhibition zone was measured using the cross-measurement method.

#### 2.3.3 Growth curve

Bacterial growth curves were determined using a microbial growth curve analyzer with minor modifications (El Shazely et al., 2020). Overnight-grown cultures of *S. aureus* (ATCC 6538) grown in Luria-Bertani (LB) broth at 37 °C were collected and diluted with fresh LB to an optical density at 600 nm (OD_600_) of 0.8, and yeast extract was added to attain a final concentration of approximately 1 × 10^6^ CFU/mL. 100.0 mg of PSPE and HHP-PSPE powder was accurately weighed and fully dissolved in 1.0 mL of sterile PBS solution (pH 7) to prepare the antibacterial solution (100.0 mg/mL). Subsequently, 270.0 μL of LB broth, 30.0 μL of antibacterial solution or sterile PBS solution (pH 7), and 3.0 μL of bacterial suspension were sequentially dispensed into a honeycomb culture plate. After mixing, the plate was inserted into the growth cur*v*e analyzer (Bioscreen C, Oy Growth Curves Ab Ltd., Finland) for continuous monitoring. Instrument parameters were set as follows: temperature of 37 °C, fast shaking speed, detection interval of 1 h, and total running time of 24 h. The assay was terminated when the negative control bacteria reached the stationary phase. Growth curves were constructed using OD₆₀₀ values recorded at each temporal interval.

### 2.4 Free amino acids

The free amino acid content was determined according to a previously reported method with minor modifications (Zhang et al., 2020). 600.0 mg of PSPE and HHP-PSPE was fully dissolved in 25.0 mL of 5.0% (w/v) trichloroacetic acid. The mixture was sonicated for 20 min using an ultrasonic device (SCIENTZ-IID, Ningbo Scientz Biotechnology Co., Ltd., China), allowed to stand for 2 h at 4 °C in a refrigerator (GR-A2078DSF, LG Electronics Inc., South Korea), and then filtered through qualitative filter paper to remove denatured precipitated proteins. The filtrate was centrifuged at 17,000×*g* for 30 min with a centrifuge (MGL-16MA, Meiruike Instrument (Shanghai) Co., Ltd., China), and the resulting supernatant was analyzed using an automatic amino acid analyzer (Hitachi L-8800, Hitachi, Ltd., Tokyo, Japan).

### 2.5 Ultraviolet-visible (UV–vis) spectroscopy

UV–Vis diffuse reflectance spectra were recorded using a spectrophotometer (UV-3600Plus, Shimadzu, Kyoto, Japan) over the wavelength range of 200–400 nm with minor modifications (Fadimu et al., 2021). PSPE and HHP-PSPE powder were uniformly ground and compacted into a dedicated sample cell to form a flat and smooth sample surface with consistent loading thickness for all groups. A standard barium sulfate (BaSO_4_) cell was employed as the baseline reference. Subsequently, the BaSO_4_ reference cell was removed from the integrating sphere and replaced with a sample cell containing compacted test samples.

### 2.6 Fourier transform infrared (FTIR) spectroscopy

The sample of FTIR spectroscopy was determined using an FTIR spectrometer (PerkinElmer, USA) with minor modifications (Zhang et al., 2024). PSPE and HHP-PSPE powder and potassium bromide (KBr) were dried in an oven (DHG-9240A, Shanghai Yiheng Technology Instrument Co., Ltd.,‌‌‌ China) at a constant temperature of 105 °C for 12 h to completely remove residual moisture. 10.0 mg of powder was weighed and fully ground with 500.0 mg of pre-dried spectroscopic-grade KBr powder in an agate mortar under dry and low-humidity conditions until the mixture presented uniform fine particles, ensuring full homogeneity of the sample-KBr mixture. Subsequently, the homogeneous mixture was placed in a dedicated mold and pressed into transparent, uniform thin tablets under a certain pressure for FTIR detection. All FTIR spectra were recorded using an FTIR spectrometer (PerkinElmer, USA) over a wavenumber range of 4000–400 cm^−1^ with a spectral resolution of 4 cm^−1^. The background spectrum of a pure KBr tablet was subtracted before sample testing.

### 2.7 Free sulfhydryl groups and disulfide bonds

Free sulfhydryl and disulfide bond contents were determined using Ellman's reagent (DTNB) with minor modifications (Zhang, Song, et al., 2022). 15.0 mg of PSPE and HHP-PSPE was dissolved in 5.0 mL of Tris-Gly buffer (0.086 M Tris, 0.09 M glycine, 4 mM EDTA, pH 8.0). After adding 50.0 μL of DTNB reagent, the mixture was vortexed and incubated at room temperature for 1 h. After incubation, the solution was centrifuged at 6000 ×*g* for 10 min with a centrifuge (MGL-16MA, Meiruike Instrument (Shanghai) Co., Ltd., China), and the absorbance of the supernatant was measured at 412 nm using a microtiter plate reader (Spark™ 10 M, Tecan Group Ltd., Switzerland). A blank control was prepared under identical experimental conditions without the addition of the DTNB reagent. For total sulfhydryl determination, samples were dissol*v*ed in Tris-Gly buffer containing 8 M urea, and the subsequent procedure was identical to that used for free sulfhydryl analysis:

$${\mathrm{SH}}_{T}-{\mathrm{SH}}_{F}\;\left(\text{μmol}/g\right)=\left(75.53\times A_{412}\times D\right)/C$$

$$-S-S-\left(\text{μmol}/g\right)=\left({\mathrm{SH}}_{T}-{\mathrm{SH}}_{F}\right)/2$$

(Note: SH_T_: Content of total sulfhydryl; SH_F_: Content of free sulfhydryl; -S-S-: Content of disulfide bond; 73.53: The molar extinction coefficient of Ellman's reagent (L·mol^−1^·cm^−1^); A₄₁₂: The absorbance measured at a wavelength of 412 nm; D: The dilution factor; C: The sample concentration (mg/mL).)

### 2.8 Molecular weight determination

Molecular weight distribution was determined by size-exclusion high-performance liquid chromatography (SEC-HPLC) with minor modifications (Li, Xie, et al., 2025). SEC-HPLC analysis was performed to determine molecular weight distributions with an Agilent 1260 HPLC system (Agilent Technologies Co., Ltd., California, USA), equipped with a RI detector and a Waters Ultrahydrogel column (300 × 7.8 mm, mixed-bed 500/250/120 Å). PSPE and HHP-PSPE powder were dissolved in 0.1 M NaNO_3_ at concentrations of 0.05–0.5% (*w*/*v*) and filtered through a 0.45 μm membrane filter. Subsequently, aliquots of 20.0 μL were injected into the chromatographic system, using 0.1 M NaNO_3_ as the mobile phase at a flow rate of 1.0 mL/min, with separation at 30 °C. A standard curve was established using polyethylene glycol (PEG) as a molecular weight standard following serial dilution, and the molecular weight of the samples was calculated accordingly.

### 2.9 Surface hydrophobicity

Surface hydrophobicity (H_0_) was determined using 1-anilino-8-naphthalenesulfonic acid (ANS) as a fluorescent probe with minor modifications (Liu et al., 2024). PSPE and HHP-PSPE powder were fully dissolved in 10.0 mmol/L PBS (pH 7.0), and serially diluted to concentrations at 0.06, 0.15, 0.6, 1.2 and 3.6 mg/ml. For each diluted sample solution, 2.0 mL of sample solution was mixed with 20.0 μL of 8.0 mmol/L ANS reagent and incubated in the dark for 15 min. Fluorescence intensity was measured using a Hitachi FLS-980 spectrofluorometer (Hitachi Ltd., Tokyo, Japan). The detection parameters were set as follows: excitation wavelength of 39.0 nm, emission wavelength of 470 nm, and both excitation and emission slit widths of 5.0 nm. Blank control groups without ANS reagent were synchronously tested to eliminate the inherent fluorescence interference of the sample buffer system. H_0_ was calculated as the initial slope of the linear regression curve of fluorescence intensity versus protein concentration.

### 2.10 Zeta potential and particle size analysis

Zeta potential and particle size were determined using a nanoparticle size analyzer (Model ZS-90, Malvern Instruments, UK) with minor modifications (Hei et al., 2024). 1.0 mg of PSPE and HHP-PSPE were fully dissolved in 10.0 mL deionized water to a concentration of 0.1 mg/mL, and then sonicated for 5 min using an ultrasonic device (SCIENTZ-IID, Ningbo Scientz Biotechnology Co., Ltd., China). Subsequently, 1.0 mL of the test solution was transferred into a standard zeta potential cuvette, and the cuvette was placed into the detection chamber of the nanoparticle size analyzer for testing. The refractive index of the medium was set to 1.33. Zeta potential and particle size were then measured at 25 °C. All measurements were performed in triplicate, and the average values were calculated.

### 2.11 Data analysis

Experimental data were processed and visualized using OriginPro 2018C software (OriginLab Corporation, Northampton, MA, USA). One-way analysis of variance (ANOVA) was conducted using IBM SPSS Statistics 23.0 software (IBM Corp., Armonk, NY, USA). Significant differences among mean values were determined by Duncan's multiple range test at a significance level of *p* < 0.05. Pearson's correlation analysis was performed between structural characteristics, physicochemical properties, and antibacterial properties and plotted using OriginPro 2018C. All data are expressed as means ± standard deviations (SD).

## 3 Results and discussion

### 3.1 Degree of hydrolysis

DH is a critical indicator for evaluating the extent of peptide bond cleavage during enzymatic hydrolysis, which directly reflects the hydrolysis efficiency and structural fragmentation degree of protein substrates (Gharehbeglou et al., 2024). The DH of PSPE can be used to reflect the enzymatic hydrolysis efficiency of proteins modified by HHP.

**Fig. 1:**
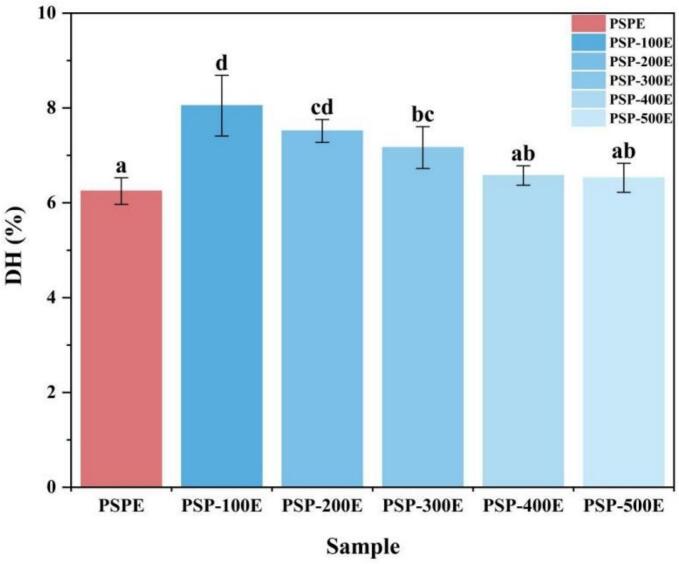
Degree of hydrolysis of the sample treated by HHP-assisted enzymic hydrolysis. (Note: PSPE, PSP-100E, PSP-200E, PSP-300E, PSP-400E, and PSP-500E indicate that the PSP were treated by HHP-assisted enzymic hydrolysis at 0.1 MPa, 100 MPa, 200 MPa, 300 MPa, 400 MPa and 500 MPa, respectively. Different lowercase letters indicate significant differences among groups for the same sample (*P* < 0.05).)

As shown in Fig. 1, HHP-PSPE exhibits a higher DH than PSPE, which indicated that HHP improves the hydrolysis efficiency of PSPE. This can be attributed to HHP-induced protein unfolding, which exposes more enzymatic cleavage sites and accelerates enzymatic hydrolysis (Zhang & Mu, 2017). The DH of HHP-PSPE gradually increased and then decreased with increasing pressure. The DH of PSP-100E reached the maximum value, which was 28.84% higher than that of the control group. With further increases in pressure (200–500 MPa), the DH showed a downward trend. The underlying mechanism may be related to protein aggregation induced by higher HHP treatment (Al-Ruwaih et al., 2019). This trend is similar to a previous study on HHP-assisted enzymatic hydrolysis of soybean protein. A 19.2% rise in DH was observed under optimal processing conditions (300 MPa, 5 min), while DH decreased with further pressure elevation (Guan et al., 2018).

### 3.2 Inhibition properties

#### 3.2.1 Inhibition rate and inhibition zone

The bacterial inhibition rate and inhibition zone diameter are intuitive and reliable indices to evaluate the antibacterial ability of antibacterial substances, which can quantitatively reflect the inhibitory effect of samples on the growth and reproduction of *S. aureus* (Chen, Sun, et al., 2025; Zhao et al., 2020). In this study, these two indices were adopted to change the antibacterial activity of PSPE hydrolysates due to HHP pretreatment.

**Fig. 2:**
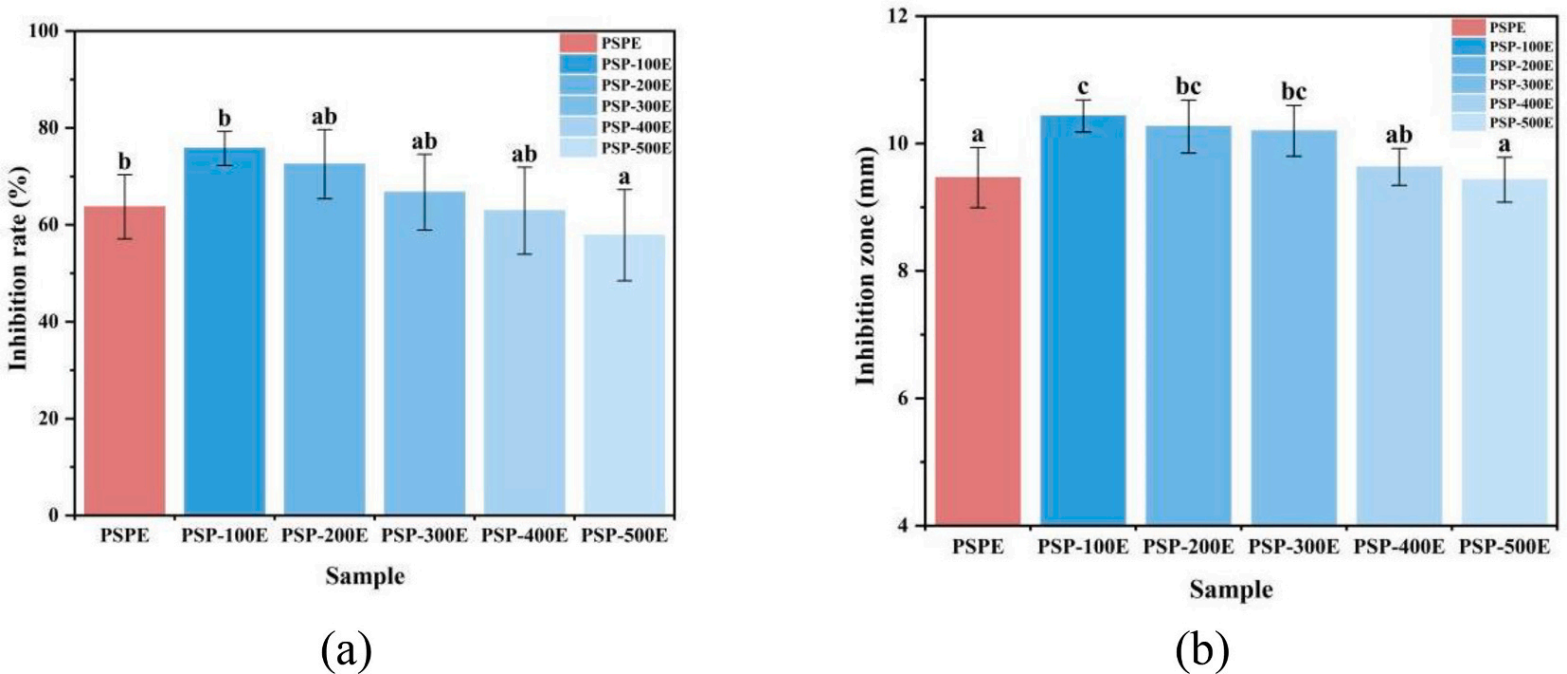
Inhibition rate (a) and inhibition zone (b) of the sample treated by HHP-assisted enzymic hydrolysis. (Note: PSPE, PSP-100E, PSP-200E, PSP-300E, PSP-400E, and PSP-500E indicate that the PSP were treated by HHP-assisted enzymic hydrolysis at 0.1 MPa, 100 MPa, 200 MPa, 300 MPa, 400 MPa and 500 MPa, respectively. Different lowercase letters indicate significant differences among groups for the same sample (*P* < 0.05).)

As presented in Fig. 2, after a 12-h incubation period, the inhibition rates and inhibition zone diameters of HHP-PSPE under 100–400 MPa were observed to be significantly elevated relative to those of PSPE, with increases ranging from 1.8% to 10.25% and from 1.27% to 18.90%, respectively. PSP-100E displayed the strongest antibacterial efficacy, characterized by enhancements of 10.25% in inhibition rate and 18.90% in inhibition zone diameter. However, antibacterial activity gradually declined as the treatment pressure exceeded 100 MPa, which was similar to the trend observed for DH.

#### 3.2.2 Growth curve

**Fig. 3:**
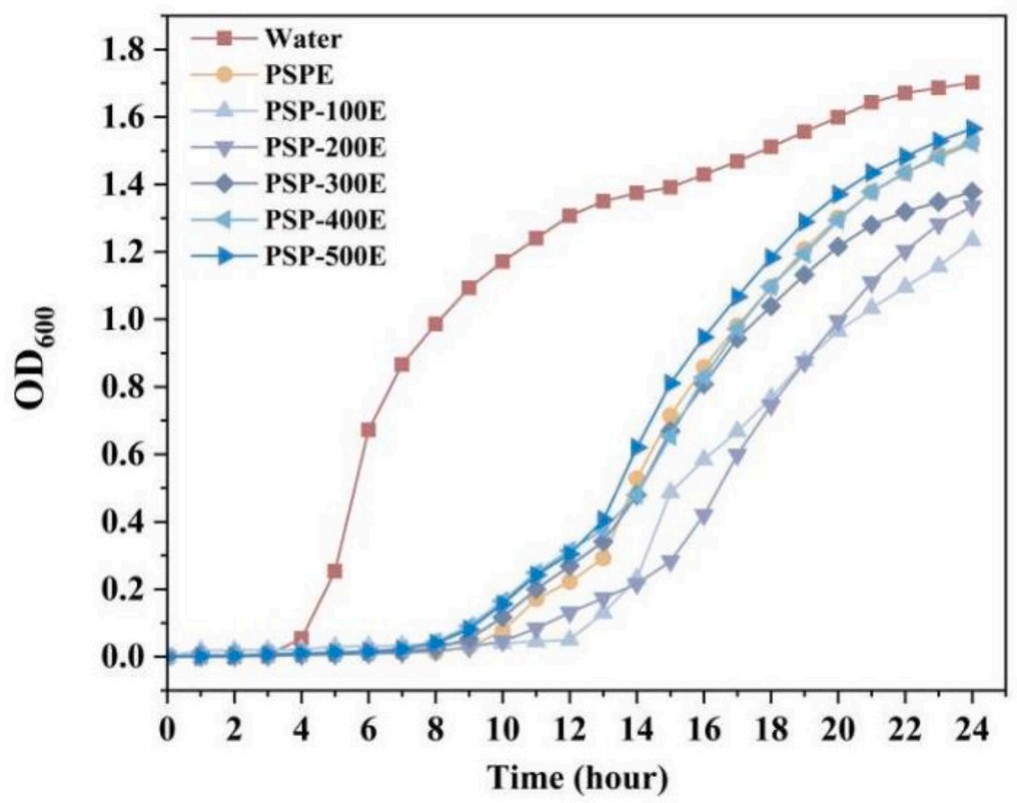
Growth curve of the sample treated by HHP-assisted enzymic hydrolysis. (Note: PSPE, PSP-100E, PSP-200E, PSP-300E, PSP-400E, and PSP-500E indicate that the PSP were treated by HHP-assisted enzymic hydrolysis at 0.1 MPa, 100 MPa, 200 MPa, 300 MPa, 400 MPa and 500 MPa, respectively. Different lowercase letters indicate significant differences among groups for the same sample (*P* < 0.05).)

Bacterial growth curves can reflect the effects of antibacterial samples on different growth stages of microorganisms, including the lag phase, exponential phase, and stationary phase (Tincho et al., 2020). As shown in Fig. 3, PSPE and HHP-PSPE (100–500 MPa) could inhibit the growth of *S. aureus* by prolonging the lag phase and delaying the initiation of the exponential growth phase. Addition with PSPE or HHP-PSPE delayed the lag phase of *S. aureus* from 4 h to 8–13 h, indicating that the PSPE and HHP-PSPE effectively retarded bacterial growth. Meanwhile, the onset of the logarithmic phase was delayed by approximately 62.5%, 37.5%, and 25% for PSP-100E, PSP-200E, and PSP-300E compared to PSPE, respectively. Moreover, the OD_600_ values of PSP-100E, PSP-200E, and PSP-300E were consistently lower than those of PSPE, suggesting stronger inhibitory effects on *S. aureus* proliferation. These effects are attributed to the antibacterial components within HHP-PSPE, which impede the metabolic synthesis required for cell division initiation, thereby inhibiting bacterial proliferation and reducing viability (Benfield & Henriques, 2020; Bertrand, 2019). And HHP facilitates the enrichment of functional substances that disrupt these physiological processes, resulting in substantial inhibition of *S. aureus* growth.

Comprehensive analysis of DH and antibacterial properties revealed that HHP within an appropriate pressure range enhances the bioactivity of PSPE. The underlying mechanism may involve pressure-induced elevation of DH, which promotes the generation of antibacterial substances (Al Saiqali et al., 2018). Conversely, excessive pressure induced protein aggregation, which impeded enzyme-substrate interactions to reduce DH and with lower antibacterial effect.

### 3.3 Free amino acids

Free amino acid content and composition are important supplementary indices for evaluating enzymatic hydrolysis efficiency and antibacterial activity, which are more indicative than total amino acid content in reflecting the structural changes and functional differences of enzymatic hydrolysates (Kim, 2017). Analyzing the variation of free amino acids under different HHP pressures (100–500 MPa) is conducive to revealing the mechanism of pressure-regulating hydrolysis reactions and antibacterial performance.

**Fig. 4:**
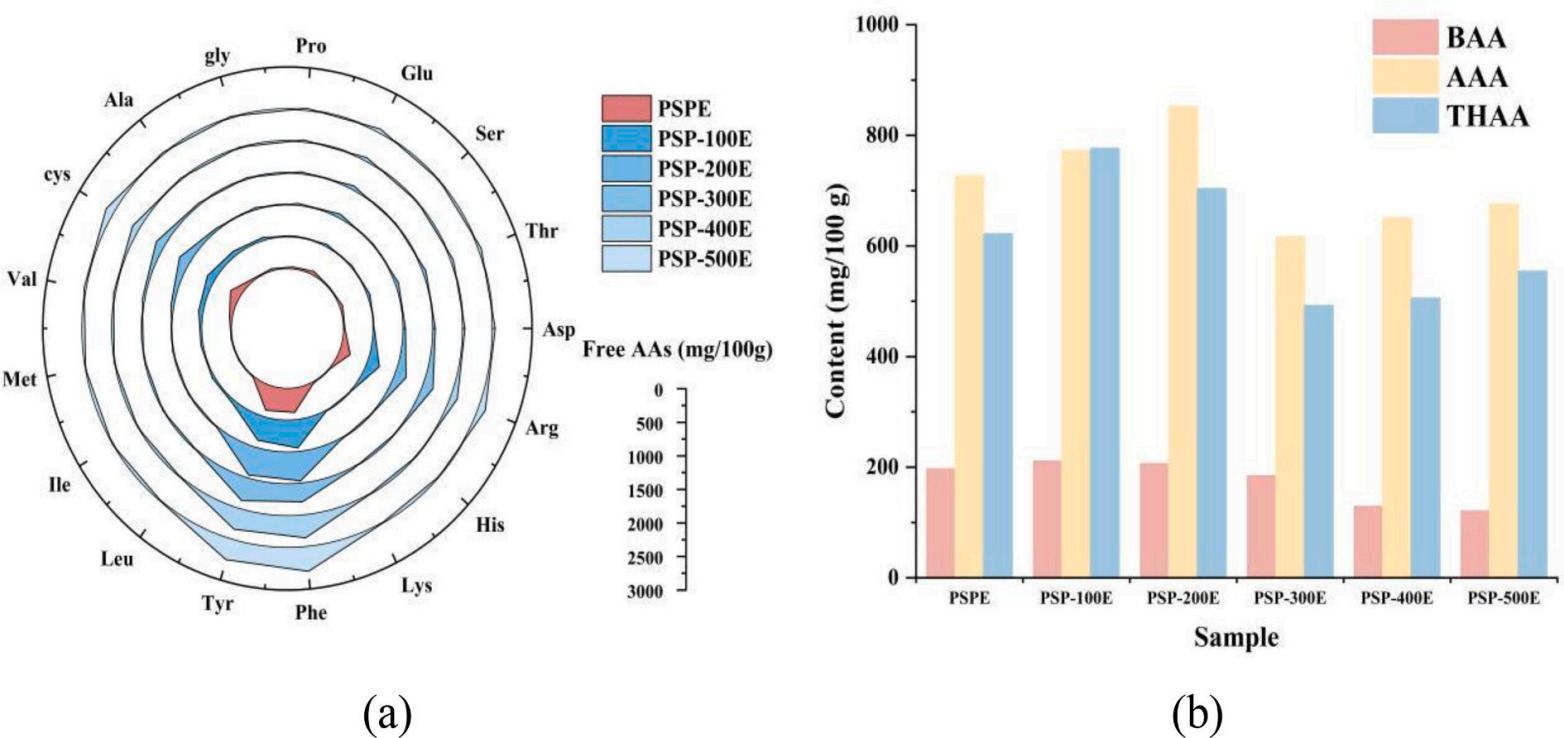
Free amino acid content (a) and the main related antibacterial component content (b) of the sample treated by HHP-assisted enzymic hydrolysis. (Note: PSPE, PSP-100E, PSP-200E, PSP-300E, PSP-400E, and PSP-500E indicate that the PSP were treated by HHP-assisted enzymic hydrolysis at 0.1 MPa, 100 MPa, 200 MPa, 300 MPa, 400 MPa and 500 MPa, respectively. BAA: Basic amino acids; AAA: Aromatic amino acids; THAA: Hydrophobic amino acids. Different lowercase letters indicate significant differences among groups for the same sample (*P* < 0.05).)

As shown in Fig. 4(a), the total free amino acid contents in PSP-100E and PSP-200E were significantly higher relative to PSPE, exhibiting increases of approximately 9.25% and 12.54%, respectively. These results indicate that HHP effectively enhances free amino acid levels in PSPE. The underlying mechanism can be attributed to the unfolding and improved solubility of PSP induced by HHP, which enhances papain accessibility to peptide bonds and accelerates the liberation of free amino acids (Franck et al., 2019; Kim, 2017). This phenomenon is consistent with the result of HHP-assisted enzymatic hydrolysis of wheat gluten hydrolysate, which increased total free amino acids from 222.0 for the blank to 29,297.0 mg/kg than the untreated sample and with higher solubility (Kim, 2017).

Nevertheless, the total free amino acid content of HHP-PSPE decreased significantly under the pressure exceeded 300 MPa. This change may be caused by excessive pressure-induced PSP intermolecular cross-linking and soluble aggregation. The aggregated PSP structure would shield the enzymatic hydrolysis sites, hinder the progress of the hydrolysis reaction, and reduce the release of free amino acids (de Carvalho Oliveira et al., 2024). In addition, papain used in this study is an endopeptidase that mainly cleaves internal peptide bonds and primarily produces peptides rather than free amino acids (Tacias-Pascacio et al., 2020). Thus, excessive pressure induced structural aggregation and reduced enzymatic accessibility, and finally led to the decrease in free amino acid content.

Antibacterial activity is closely related to the composition of functional free amino acids. Basic amino acids (BAA), aromatic amino acids (AAA), and hydrophobic amino acids (THAA) can synergize with antibacterial peptides to destroy bacterial membrane structure and enhance antibacterial effect (Li et al., 2022; Tong et al., 2014). Consequently, those free amino acids are essential indicators to elucidate the underlying mechanisms of changes in antibacterial activity.

As presented in Fig. 4b, PSP-100E and PSP-200E exhibited significantly higher BAA, AAA, and THAA contents than the control group, with increases of 7.04%, 6.33%, 24.85% and 4.67%, 17.32%, 13.08%, respectively. Elevated contents of BAA and AAA may exert synergistic effects with cationic antibacterial peptides on negatively charged bacterial membranes. These interactions increase membrane permeability, disrupt membrane integrity, trigger the leakage of intracellular substances, and ultimately result in bacterial death (Di Somma et al., 2020). Higher THAA contents can be inserted into lipid bilayers together with hydrophobic peptides to disrupt membrane integrity and accelerate cytoplasmic leakage (Zhang et al., 2021). These results indicated that the abundant functional amino acids in HHP-PSPE under optimized pressure formed a synergistic antibacterial system with bioactive peptides, which partially accounted for the improved antibacterial performance of HHP-PSPE. However, the specific contributions of free amino acids and their synergistic interactions with antibacterial peptides remain to be further experimentally validated.

### 3.4 UV–vis spectroscopy

UV–Vis spectroscopy is an effective technical means to characterize protein conformational changes, peptide bond exposure, and aromatic amino acid microenvironment. The absorption intensity at 200–220 nm reflects the exposure of peptide bonds, while the spectral range of 240–300 nm reflects the microenvironmental changes of Trp, Tyr, and Phe residues (Chang et al., 2017; Saraiva, 2020). Accordingly, UV–Vis spectroscopy can be used to characterize the structural reaction mechanism of PSPE under different HHP pressures.

**Fig. 5:**
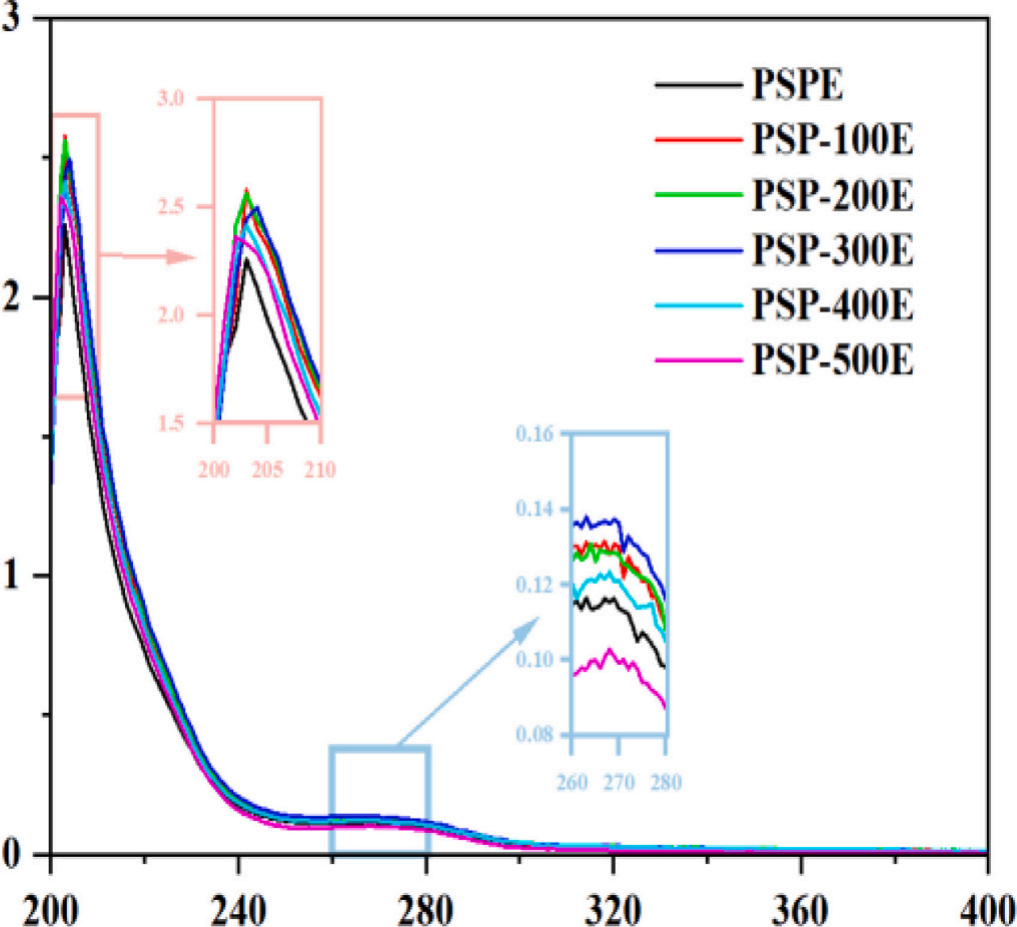
UV–Vis Spectroscopy of the sample treated by HHP-assisted enzymic hydrolysis. (Note: PSPE, PSP-100E, PSP-200E, PSP-300E, PSP-400E, and PSP-500E indicate that the PSP were treated by HHP-assisted enzymic hydrolysis at 0.1 MPa, 100 MPa, 200 MPa, 300 MPa, 400 MPa and 500 MPa, respectively.)

As shown in Fig. 5, the absorption intensity at 203 nm for HHP-PSPE under 100–500 MPa was higher than for PSPE. The stronger absorption intensity of HHP-PSPE indicated that moderate HHP induced protein unfolding to expose more peptide bonds. The loosened protein structure strengthened enzyme-substrate interactions and accelerated peptide bond cleavage to improve the degree of hydrolysis and intensity of the characteristic absorption peak (Johnson Esua et al., 2022). PSP-100E exhibited the strongest absorption peak, while absorbance gradually declined as pressure rose further. This trend may be attributed to protein refolding and reduced solubility by excessive pressure, which impede interactions between PSP and enzymes (Wang et al., 2022). These observations are similar to the results of DH.

In the 240–300 nm characteristic absorption region of aromatic amino acids, the absorption intensity of PSP-100E to PSP-400E was significantly higher than that of the control. This result showed that HHP pretreatment effectively enhanced the exposure of PSP internal aromatic amino acid residues. This similar spectral trend has also been observed in HHP-assisted enzymatic hydrolysates of soybean glycinin, which displayed higher UV absorption intensity after pressure treatment (Li, Zhao, et al., 2025). Aromatic amino acids with exposed side chains can enhance the interfacial affinity between peptide fragments and bacterial lipid membranes, which may promote the insertion of active peptides into cell membranes and improve the antibacterial efficiency (Tan et al., 2021). The similar variation trend between UV absorption characteristics and antibacterial activity reveals that aromatic amino acid residues of peptides may constitute one potential factor for affecting the antimicrobial performance of hydrolysates.

### 3.5 FTIR spectroscopy

The correlation between the secondary structure of antibacterial peptides and their membrane-binding behavior has been well explored (Zhang, Xu, & Dong, 2022). In this study, Fourier transform infrared (FTIR) spectroscopy was used to characterize pressure-induced changes in the secondary structure of PSPE. This further helped to reveal the underlying mechanism for the improved antibacterial activity induced by HHP.

**Fig. 6:**
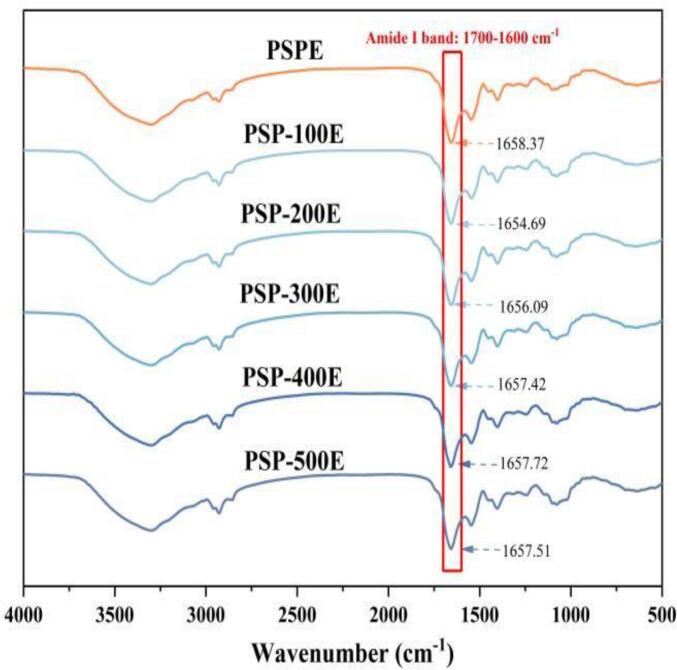
FTIR spectroscopy of the sample treated by HHP-assisted enzymic hydrolysis. (Note: PSPE, PSP-100E, PSP-200E, PSP-300E, PSP-400E, and PSP-500E indicate that the PSP were treated by HHP-assisted enzymic hydrolysis at 0.1 MPa, 100 MPa, 200 MPa, 300 MPa, 400 MPa and 500 MPa, respectively.)

As shown in Fig. 6, no new characteristic peaks appeared in the spectra of HHP-PSPE under 100–500 MPa, which indicated that no new chemical functional groups or covalent complexes were formed after HHP-assisted enzymatic hydrolysis. The structural changes observed were mainly derived from non-covalent rearrangement. The band at 1600–1700 cm^−1^ represents the stretching vibration of C=O (amide I band). The characteristic peak at this band of HHP-PSPE under 100–500 MPa shifted from 1658.37 cm^−1^ (PSPE) to 1654.69–1657.72 cm^−1^, indicating that the secondary structure of PSPE was significantly influenced by HHP. This phenomenon can be attributed to the higher DH induced by HHP, which generates abundant short peptides in the HHP-PSPE. Those peptides spontaneously rearranged and accompanied with the increasing content of intramolecular hydrogen bonds. This reduces the stretching vibration frequency of C=O groups and ultimately induces a red shift of the FTIR amide I band (Yang et al., 2025). This result is similar to previous observations on casein hydrolysates, where increased DH corresponded to more obvious red shifts of the amide I band (Wang et al., 2013).

Peak area quantification combined with second-derivative deconvolution and Gaussian curve fitting was used to clarify how HHP pretreatment alters secondary structural fractions of PSPE and HHP-PSPE (Zhao et al., 2019). As shown in Table 1, HHP-PSPE under 100–500 MPa exhibited *α*-helix contents increased by 4.34–23.15% and *β*-sheet contents were reduced by 6.68–36.85% compared with PSPE. Previous studies reported that within an appropriate hydrolysis range, DH showed a linear positive correlation with *α*-helix contents and a negative correlation with *β*-sheet contents (Rodriguez et al., 2025). This phenomenon can be explained by preferential enzymatic hydrolysis of compact *β*-sheet regions and the resulting short peptides rearranging into flexible, extended *α*-helix structures (Shuai et al., 2022). However, this explanation and trend are not suitable for all studies. For example, HHP-assisted enzymatic hydrolysis of lentil protein isolate with higher content of *β*-sheet and lower content of *α*-helical (Ahmed et al., 2019). These discrepancies may depend on the specific enzyme and protein types (Xu et al., 2020).

**Table 1:** Secondary structure content of the sample treated with different treated conditions.

Sample	*α*-Helix	*β*-Sheet	*β*-Turn	Random coil
PSPE	23.50 ± 0.57 a	24.61 ± 0.79 e	41.16 ± 0.67 a	10.73 ± 0.31 bc
PSP-100E	28.47 ± 0.43 d	17.98 ± 0.87 a	42.80 ± 0.44 c	10.75 ± 0.01 bc
PSP-200E	26.83 ± 0.38 c	20.40 ± 0.23 b	41.68 ± 0.59 ab	11.09 ± 0.04 c
PSP-300E	26.41 ± 0.30 c	21.81 ± 0.29 c	41.21 ± 0.38 a	10.57 ± 0.26 b
PSP-400E	24.69 ± 0.39 b	22.46 ± 0.25 cd	43.40 ± 0.55 c	10.42 ± 0.26 b
PSP-500E	24.52 ± 0.37 b	23.07 ± 0.20 d	42.37 ± 0.63 bc	10.03 ± 0.13 a

(Note: PSPE, PSP-100E, PSP-200E, PSP-300E, PSP-400E, and PSP-500E indicate that the PSP were treated by HHP-assisted enzymic hydrolysis at 0.1 MPa, 100 MPa, 200 MPa, 300 MPa, 400 MPa and 500 MPa, respectively. Different lowercase letters indicate significant differences among groups for the same sample (*P* < 0.05).)

Collectively, these results indicated that HHP-assisted enzymatic hydrolysis may increase the generation of short peptides, which tend to self-assemble into *α*-helix structures (Li et al., 2022). The elevated *α*-helix content may facilitate structural rearrangement during contact with bacterial membranes. This rearrangement drives the separation of hydrophilic and hydrophobic residues to form an amphipathic structure. Such a structure could insert into lipid bilayers and form transmembrane pores to disrupt bacterial membrane integrity (Ma et al., 2024). Therefore, these structural changes may partially account for the superior antibacterial activity of HHP-PSPE compared with PSPE.

### 3.6 Free sulfhydryl groups and disulfide bonds

Free sulfhydryl (-SH) and disulfide bond (-S-S-) represent critical covalent linkages sustaining the structural stability of proteins (Liu, Pan, et al., 2025). These dynamic variations reflect the structural stability of PSP treated by HHP-assisted enzymatic hydrolysis at pressures ranging from 100 to 500 MPa.

**Fig. 7:**
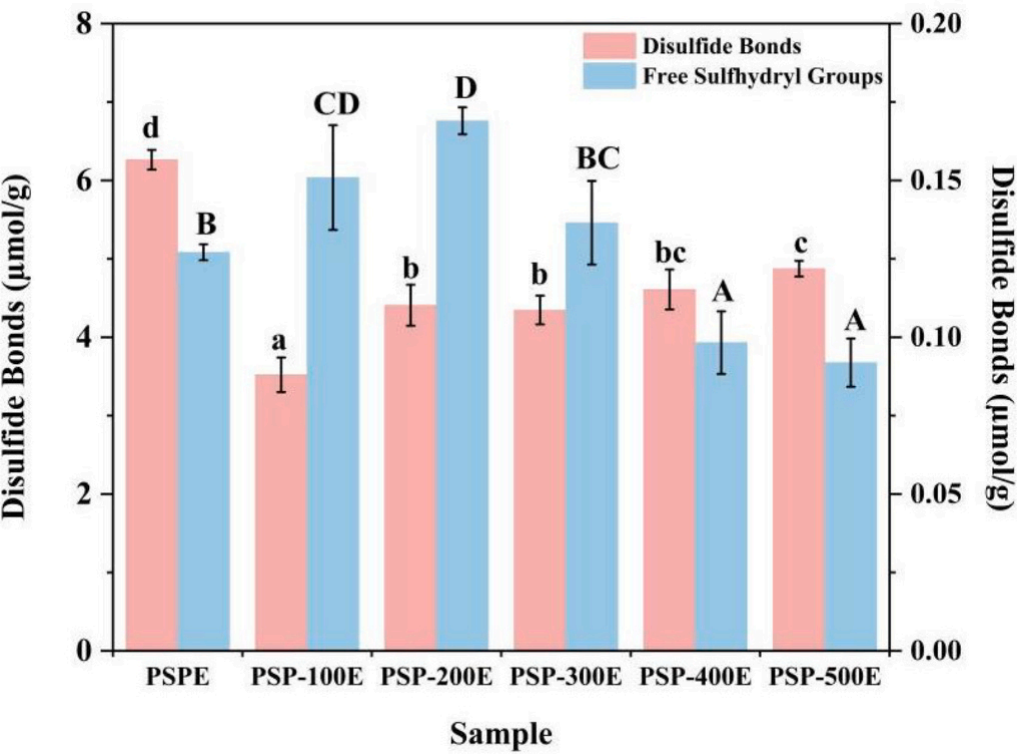
Free sulfhydryl groups and disulfide bonds content of the sample treated by HHP-assisted enzymic hydrolysis. (Note: PSPE, PSP-100E, PSP-200E, PSP-300E, PSP-400E, and PSP-500E indicate that the PSP were treated by HHP-assisted enzymic hydrolysis at 0.1 MPa, 100 MPa, 200 MPa, 300 MPa, 400 MPa and 500 MPa, respectively. Different letters meant there was significant difference among groups within same sample (*P* < 0.05).)

As shown in Fig. 7, the -S-S- content of HHP-PSPE under 100–500 MPa exhibited a reduction of 28.51–77.56% compared with PSPE. Meanwhile, the free -SH content of HHP-PSPE under 100–300 MPa exhibited a significant elevation of 7.40–32.97% relative to PSPE. HHP induced protein conformational changes by disrupting non-covalent interactions, which promotes PSPE unfolding and structural expansion. Furthermore, the dissociation of protein subunits and the cleavage of -S-S- expose buried internal sulfhydryl groups, elevating the content of free -SH (Lyu et al., 2023; Wang, Wang, et al., 2021). This increase in free -SH correlates with reduced protein rigidity and enhanced solubility, which are critical factors for improved functional properties (Liu, Pan, et al., 2025). Accordingly, uniform dispersion of HHP-PSPE in bacterial suspensions is improved, which increases its probability to interact with bacterial cell membranes (Rao et al., 2024). In contrast, PSP-400E and PSP-500E exhibited higher -S-S- content and lower free -SH content. This phenomenon was attributed to the re-oxidation of free -SH into disulfide bonds facilitated by excessive pressure, which could induce protein reaggregation to decrease enzymatic hydrolysis efficiency (Yin et al., 2009). The results are similar to previous findings on rice bran protein hydrolysates, which reported that free -SH content declined with increasing pressure and was lower than that of the control (Wang, Wang, et al., 2021).

### 3.7 Molecular weight determination

Molecular weight distribution is considered a critical indicator for evaluating the degree of protein hydrolysis, which can effectively characterize the synergistic effect of HHP on the enzymatic hydrolysis of PSP (Yolandani et al., 2023).

**Fig. 8:**
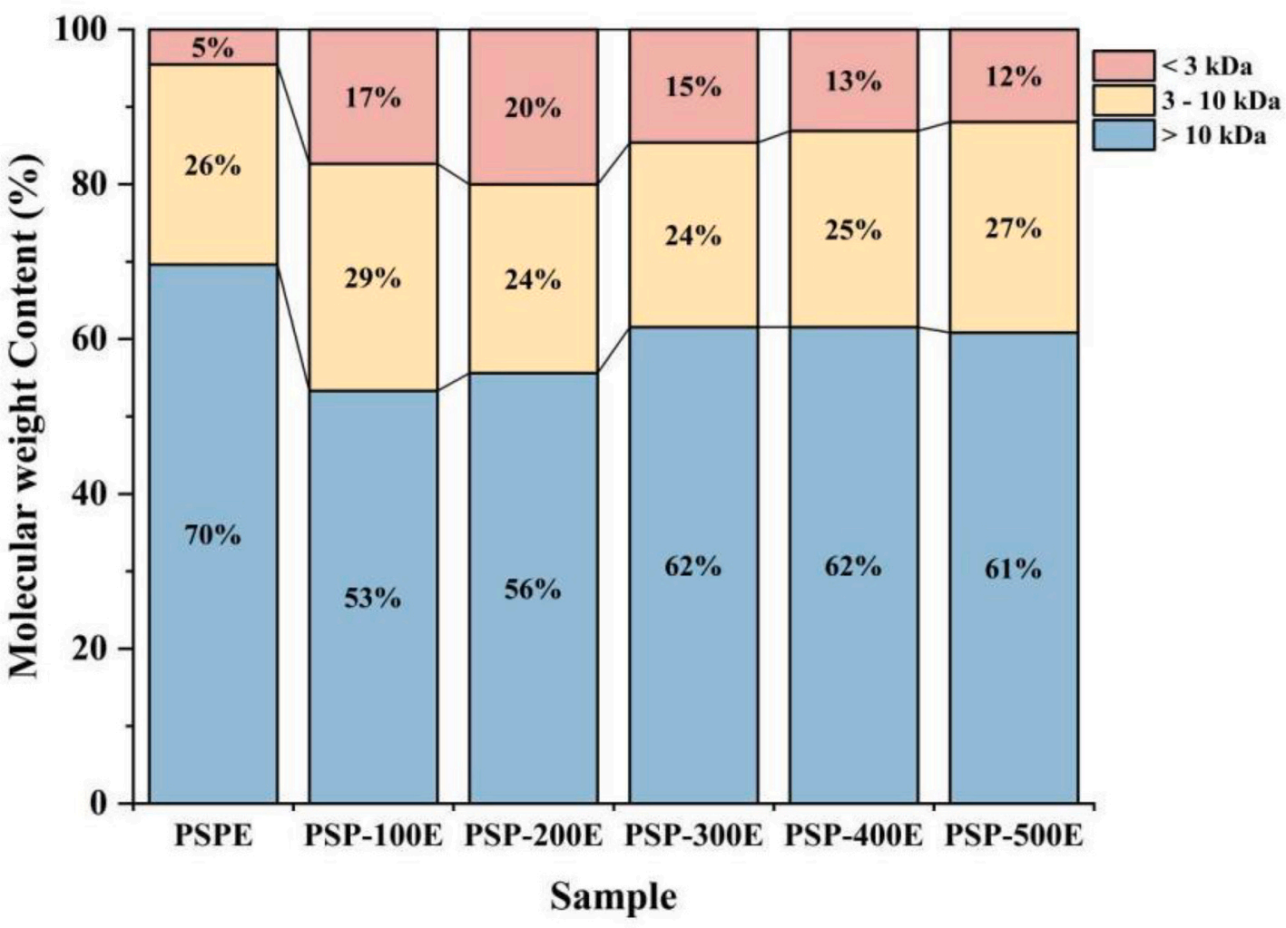
Molecular weight determination of the sample treated by HHP-assisted enzymic hydrolysis. (Note: PSPE, PSP-100E, PSP-200E, PSP-300E, PSP-400E, and PSP-500E indicate that the PSP were treated by HHP-assisted enzymic hydrolysis at 0.1 MPa, 100 MPa, 200 MPa, 300 MPa, 400 MPa and 500 MPa, respectively.)

As shown in Fig. 8, HHP-PSPE showed significant reduction in macromolecular fractions (>10 kDa) and a substantial increase in small molecular peptide fractions (<10 kDa). The relative proportion of small peptides in HHP-PSPE was 22.58–48.39% higher than that in native PSPE. Peptides smaller than 3 kDa displayed a significant increase of 185.71% relative to the control. This indicates that HHP facilitates enzyme cleavage of macromolecular proteins to generate abundant low-molecular-weight peptides, and this change is consistent with the trend of DH. Previous studies also reported a similar trend in sweet potato protein hydrolysates under pressure. The proportion of peptide fractions <3 kDa significantly increased from 28.5% at 0.1 MPa to 50.89% at 300 MPa (Zhang & Mu, 2017).

Additionally, some research has reported that proteins with high molecular weight limit structural flexibility, while those with lower molecular weight tend to exhibit enhanced functional properties and bioactivity (including antibacterial activity) (Osman et al., 2021; Yolandani et al., 2023). Therefore, this finding indicates that the different content in lower molecular weight between HHP-PSPE and PSPE could explain their distinct antibacterial activities.

### 3.8 Hydrophobicity

**Fig. 9:**
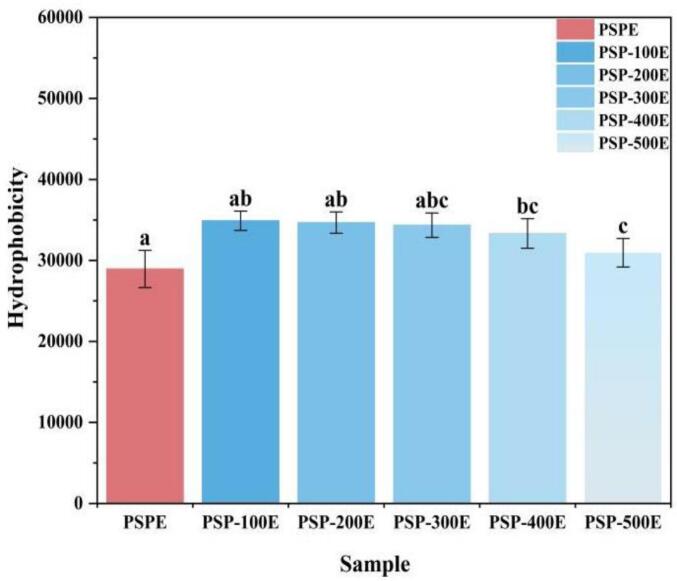
Hydrophobicity of the sample treated by HHP-assisted enzymic hydrolysis. (Note: PSPE, PSP-100E, PSP-200E, PSP-300E, PSP-400E, and PSP-500E indicate that the PSP were treated by HHP-assisted enzymic hydrolysis at 0.1 MPa, 100 MPa, 200 MPa, 300 MPa, 400 MPa and 500 MPa, respectively. Different letters meant there was significant difference among groups within same sample (*P* < 0.05).)

The fluorescent probe ANS specifically binds to the hydrophobic regions of protein molecules, and the corresponding fluorescence intensity is positively correlated with protein surface hydrophobicity (Shao et al., 2024). As shown in Fig. 9, the surface hydrophobicity of HHP-PSPE under 100–400 MPa was significantly higher than that of PSPE, and PSP-100E exhibited the largest increase of 20.50%. These results indicated that HHP assisted enzymatic hydrolysis treatment effectively promoted the exposure of internal hydrophobic groups. This phenomenon can be attributed to HHP-induced unfolding of PSP, which exposes buried hydrophobic residues on the protein surface. Subsequently, HHP-treated PSP were subjected to enzymatic hydrolysis, which degraded intact macromolecules into short peptides. This process further promoted the exposure of internal hydrophobic residues and elevated the surface hydrophobicity of the hydrolysates (Jiang et al., 2023; Tong et al., 2022). In addition, the exposed hydrophobic groups could effectively bind and insert into the bacterial phospholipid bilayer, disrupting the structural balance of cell membranes. This interaction induces pore formation and cytoplasmic leakage, which ultimately results in bacterial lysis (Ma et al., 2024). The similar trend between hydrophobicity and antibacterial activity revealed that the stronger hydrophobic interactions of HHP-PSPE may constitute a potential mechanism responsible for its superior antibacterial activity relative to PSPE.

### 3.9 Zeta potential and particle size

Particle size and zeta potential determine the dispersion uniformity of protein hydrolysates in aqueous solution, which directly affect the functional performance of the sample (Yang et al., 2023).

**Fig. 10:**
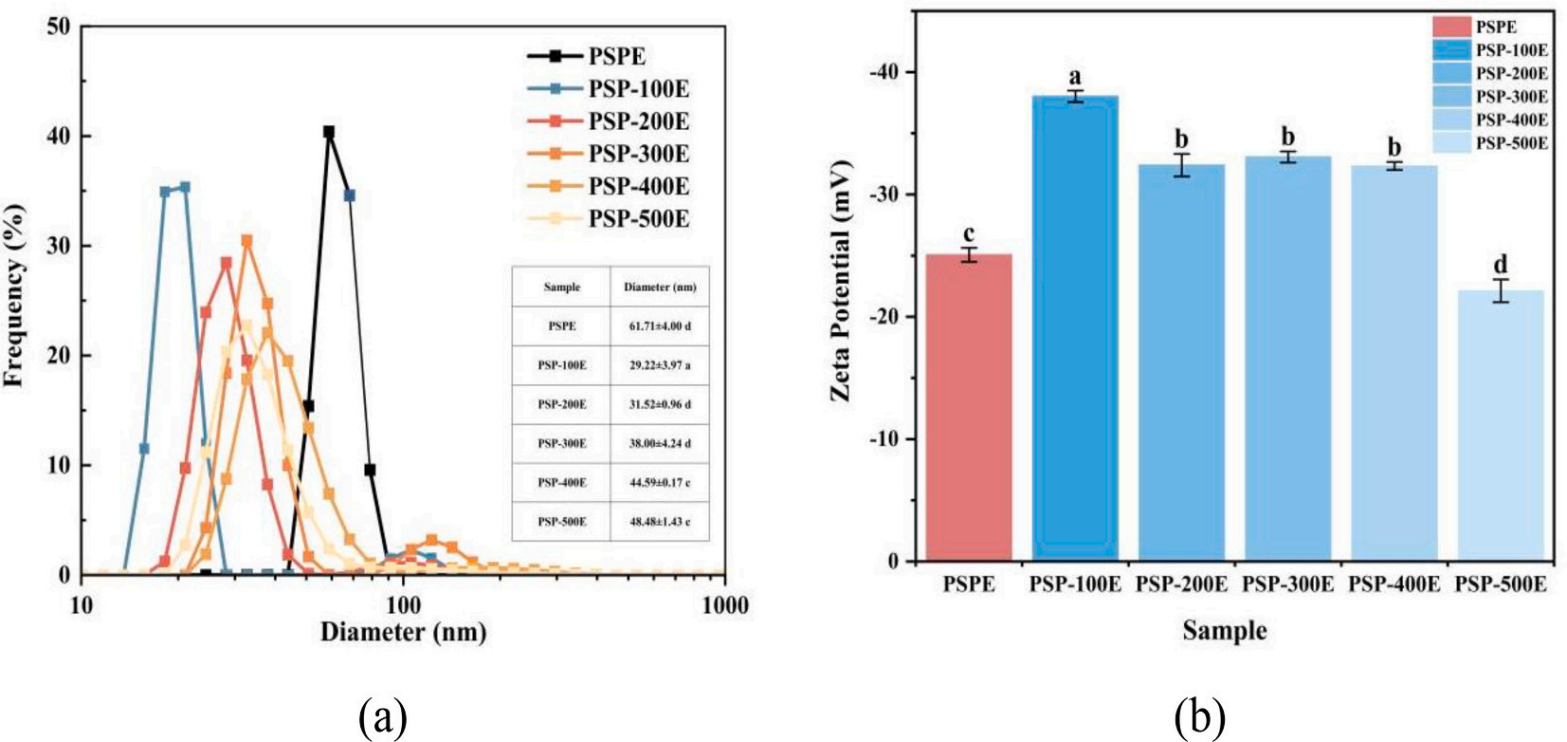
Zeta Potential (a) and particle size (b) of the sample treated by HHP-assisted enzymic hydrolysis. (Note: PSPE, PSP-100E, PSP-200E, PSP-300E, PSP-400E, and PSP-500E indicate that the PSP were treated by HHP-assisted enzymic hydrolysis at 0.1 MPa, 100 MPa, 200 MPa, 300 MPa, 400 MPa and 500 MPa, respectively. Different letters meant there was significant difference among groups within same sample (*P* < 0.05).)

As shown in Fig. 10(a), the particle size of HHP-PSPE exhibited a substantial reduction, with decreases ranging from 27.29% to 111.91% compared to PSPE. This phenomenon may be attributed to the disruption of large insoluble PSP aggregates by HHP treatment accompanied by protein structural loosening. This modification enhanced protein susceptibility to enzymatic hydrolysis, thereby reducing particle dimensions (Liu, Li, et al., 2022). However, the particle size distribution of HHP-PSPE under 200–500 MPa gradually broadened as pressure increased compared with PSP-100E, which can be attributed to excessive pressure-induced PSP reaggregation (Liu, Pan, et al., 2025). Enzymatic hydrolysis efficiency may be decreased by PSP aggregation, which results in particle size distribution and poor dispersion uniformity of HHP-PSPE under 200–500 MPa than 100 MPa.

As illustrated in Fig. 10(b), PSP-100E exhibited the maximum absolute zeta potential, representing a 39.97% increase relative to PSPE. The increased absolute potential enhanced the electrostatic repulsion between particles, effectively improving the dispersibility and stability of hydrolysates in solution (Liu, Lin, et al., 2022).

Combined with the above analysis, HHP-assisted enzymatic hydrolysis within a suitable pressure range reduces PSP particle size while improving PSP stability and dispersibility. This enables protein hydrolysate to fully exert their biological functions in solution, including antibacterial activity. Meanwhile, the smaller particle size boosts the specific surface area and facilitates homogeneous dispersion of antibacterial components in solution, which increases the probability of interactions with bacterial cell membranes. Accordingly, the bioactive functional groups within hydrolysates can readily attach to target sites on bacterial surfaces, which may serve as a potential mechanism underlying the improved antimicrobial activity of HHP-PSPE (Sirelkhatim et al., 2015).

### 3.10 Correlation analysis

**Fig. 11:**
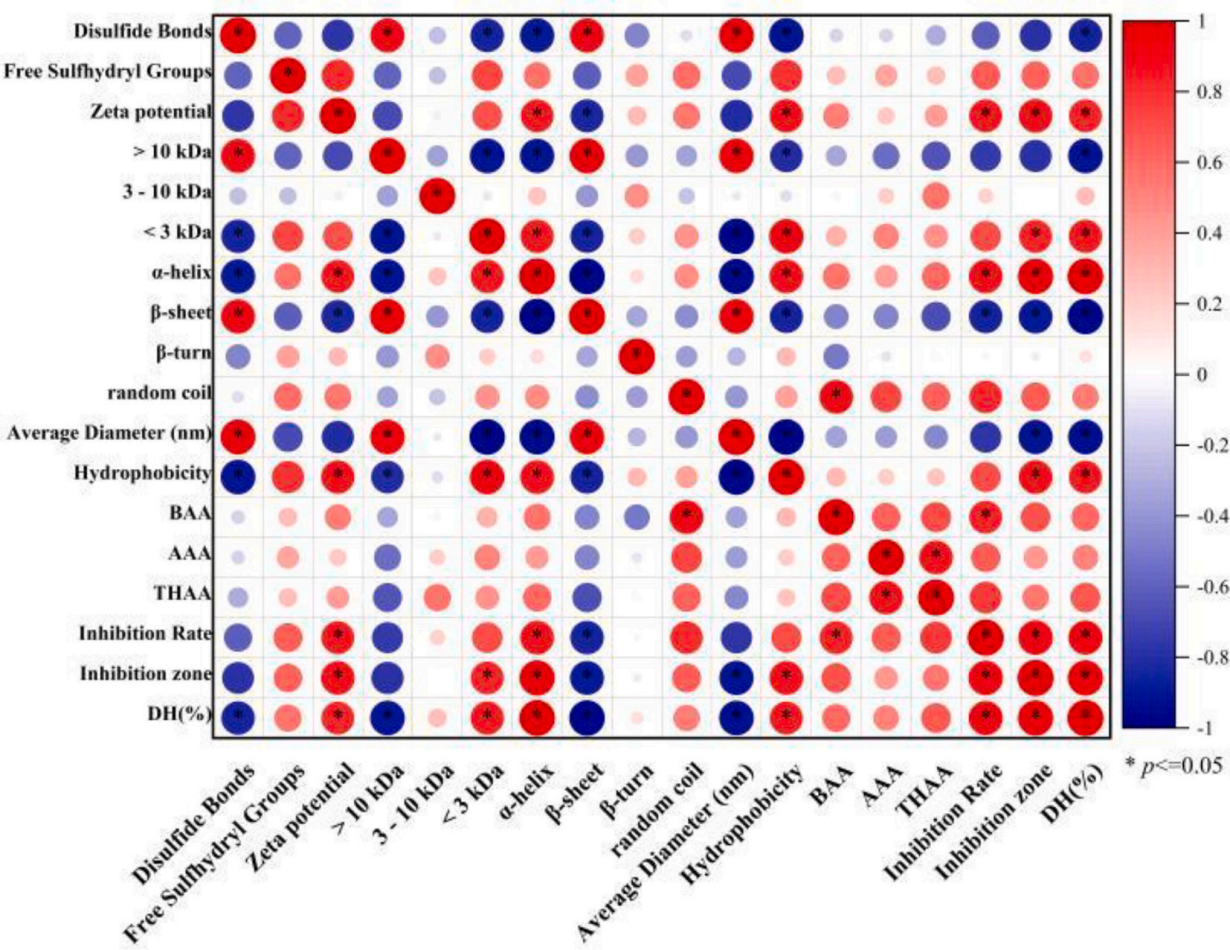
Pearson correlation between structure, physicochemical and antibacterial activity of sample. (Note: * means significant at 0.05 level.)

Pearson correlation analysis clarified the internal correlation between HHP-induced protein structure, physicochemical indices, DH and antibacterial activity. As shown in Fig. 11, DH exhibited a significant positive correlation with both the inhibition rate and the inhibition zone diameter. Zeta potential, the proportion of molecular weight fractions below 3 kDa, and *α*-helix content were also significantly positively correlated with antibacterial activity. The above results showed that HHP does not affect antibacterial activity through a single factor, but synergistically regulates protein secondary structure, molecular weight distribution, sulfhydryl/disulfide bond balance, surface hydrophobicity and solution stability. The correlation findings provide a potential theoretical basis for further clarifying the structure-physicochemical property-antibacterial activity relationship of HHP-PSPE.

## 4 Conclusion

This study developed HHP-assisted enzymatic hydrolysis as a feasible methodology designed to improve both hydrolytic efficiency and the antibacterial activity of the hydrolysate. The compact native structure of PSP was disrupted by HHP under 100–500 MPa, including protein unfolding and exposure of buried internal enzymatic cleavage sites, which strengthes enzyme - substrate interactions to increase DH. This treatment also degraded proteins into large quantities of low-molecular-weight (<3 kDa) bioactive peptides and enhanced the release of functional hydrophobic, aromatic and basic free amino acids. Furthermore, HHP-PSPE exhibited a smaller particle size, uniform particle size distribution, and higher absolute zeta potential, which indicated the improved dispersion and stability in solution. Those favorable properties create a stable and well-dispersed antibacterial microenvironment, enabling HHP-PSPE to exert its antibacterial performance more sufficiently and efficiently than PSPE. Structural characterization further revealed the potential antibacterial mechanism of HHP-PSPE. HHP changes the secondary structure of hydrolysates to increase the proportion of amphipathic *α*-helices, exposed surface hydrophobic groups and aromatic residues. These HHP-induced structural changes enables HHP-PSPE to bind to and insert into bacterial lipid bilayers, impair membrane integrity, and trigger intracellular substance leakage. Such membrane damage further suppresses the growth and proliferation of *S. aureus* by prolonging its lag phase. Among all samples, PSP-100E exhibited the optimal DH and antibacterial activity, which is closely associated with its favorable antimicrobial-related structural and physicochemical properties. However, excessive HHP pressure (>100 MPa) caused protein refolding, aggregation and sulfhydryl reoxidation, hindering hydrolysis and weakening antibacterial activity. Pearson correlation analysis also confirmed that DH, *α*-helix proportion, low-molecular-weight peptide content and stability as the pivotal potential factors responsible for the enhanced antibacterial activity.

This study systematically revealed the change rules of hydrolysis efficiency and antibacterial activity under different pressures and filled the research gap in the structure – antibacterial activity relationship of HHP-modified plant protein hydrolysates. It also provides a novel approach and theoretical basis for the high-value utilization of chili pepper seeds and resource reservoir for the development of new antibacterial peptides. However, this study still has limitations that require further in-depth exploration in future work: (1) Optimizing other equipment parameters (such as processing chamber temperature and time) and environmental conditions (solid-to-liquid ratio and pH) to systematically establish multidimensional structure-activity relationships between non-thermal treatment, plant protein structure, and enzymatic hydrolysis efficiency. (2) The HHP-PSPE of antibacterial stability under diverse environmental conditions (e.g., pH, metal ions, and temperature), cytotoxicity, hemolytic activity, and application-related functional properties, requires further research to support practical utilization. (3) On the basis of the potential antibacterial mechanism revealed in this study, subsequent studies will verify the antibacterial pathway by examining bacterial structural damage, oxidative stress, energy metabolism disturbance, and molecular and genetic variations. Comparative analyses between HHP-PSPE and PSPE will further elucidate the regulatory mechanism of HHP on antibacterial activity. Those future works can further improve the industrial application potential of HHP-PSPE, which will facilitate the large-scale development and utilization of chili seed resources.

## CRediT authorship contribution statement

**Sidi Liu:** Writing – original draft, Visualization, Validation, Software, Methodology, Investigation, Conceptualization. **Yue Han:** Methodology, Investigation. **Yiyi He:** Visualization, Investigation. **Yunkai Zhang:** Methodology. **Yu Xiong:** Visualization, Validation, Supervision. **Wenshu Huang:** Resources, Project administration. **Dong Yang:** Writing – review & editing, Supervision. **Liang Zhao:** Writing – review & editing, Validation, Supervision, Project administration, Funding acquisition, Data curation, Conceptualization. **Xiaojun Liao:** Writing – review & editing, Visualization, Validation, Resources, Conceptualization.

## Declaration of competing interest

The authors declare that they have no known competing financial interests or personal relationships that could have appeared to influence the work reported in this paper.

## Data Availability

Data will be made available on request.

## References

[bb0005] Aguilar-Toalá J.E., Deering A.J., Liceaga A.M. (2020). New insights into the antimicrobial properties of hydrolysates and peptide fractions derived from chia seed (*Salvia hispanica* L.). Probiotics and Antimicrobial Proteins.

[bb0010] Ahmed J., Mulla M., Al-Ruwaih N., Arfat Y.A. (2019). Effect of high-pressure treatment prior to enzymatic hydrolysis on rheological, thermal, and antioxidant properties of lentil protein isolate. Legume Science.

[bb0015] Al Saiqali M., Tangutur A.D., Banoth C., Bhukya B. (2018). Antimicrobial and anticancer potential of low molecular weight polypeptides extracted and characterized from leaves of *Azadirachta indica*. International Journal of Biological Macromolecules.

[bb0020] Alahmad K., Xia W., Jiang Q., Xu Y. (2022). Effect of the degree of hydrolysis on nutritional, functional, and morphological characteristics of protein hydrolysate produced from bighead carp (*Hypophthalmichthys nobilis*) using ficin enzyme. Foods.

[bib411] Al-Ruwaih N., Ahmed J., Mulla M.F., Arfat Y.A. (2019). High-pressure assisted enzymatic proteolysis of kidney beans protein isolates and characterization of hydrolysates by functional, structural, rheological and antioxidant properties. Lwt- Food Science and Technology.

[bb0025] Ana P.M.L., Hidalgo Chávez D.W., Jeane S.D.R., Mellinger-Silva C., Rosenthal A. (2021). Effect of high hydrostatic pressure on the antioxidant capacity and peptic hydrolysis of whey proteins. Ciência Rural.

[bb0030] Bandyopadhyay K., Ghosh S. (2002). Preparation and characterization of papain-modified sesame (*Sesamum indicum* L.) protein isolates. Journal of Agricultural and Food Chemistry.

[bb0035] Benfield A.H., Henriques S.T. (2020). Mode-of-action of antimicrobial peptides: Membrane disruption vs. intracellular mechanisms. Frontiers in Medical Technology.

[bb0040] Bertrand R.L. (2019). Lag phase is a dynamic, organized, adaptive, and evolvable period that prepares bacteria for cell division. Journal of Bacteriology.

[bb0045] de Carvalho Oliveira L., Martinez-Villaluenga C., Frias J., Cartea M.E., Francisco M., Cristianini M., Peñas E. (2024). Corrigendum to “High pressure-assisted enzymatic hydrolysis potentiates the production of quinoa protein hydrolysates with antioxidant and ACE-inhibitory activities” [Food Chemistry 447 (2024) 138887/ FOCH_138887]. Food Chemistry.

[bb0050] Chang C., Li X., Li J., Niu F., Zhang M., Zhou B., Su Y., Yang Y. (2017). Effect of enzymatic hydrolysis on characteristics and synergistic efficiency of pectin on emulsifying properties of egg white protein. Food Hydrocolloids.

[bb0055] Chen X., Song M., Tian L., Shan X., Mao C., Chen M., Zhao J., Sami A., Yin H., Ali U., Shi J., Li H., Zhang Y., Zhang J., Wang S., Shi C., Chen Y., Du X., Zhu K., Wu L. (2025). A plant peptide with dual activity against multidrug-resistant bacterial and fungal pathogens. *Science* Advances.

[bb0060] Chen Y., Sun J., Chen H., Yang Y., Zhang J., Wang S., Dai L. (2025). Plant-derived bioactive peptides: Extraction, isolation, purification, pharmacological activities, structure–activity relationship, and applications. Journal of Agricultural and Food Chemistry.

[bb0065] Darby E.M., Trampari E., Siasat P., Gaya M.S., Alav I., Webber M.A., Blair J.M.A. (2024). Author correction: Molecular mechanisms of antibiotic resistance revisited. Nature Reviews Microbiology.

[bb0070] Deng Z., Du X., Liu S., Xiong Y., Wang Y., Rao L., Liu M., Zhao L., Liao X. (2025). Modification of pepper seed protein isolate to improve its functional characteristic by high hydrostatic pressure [Evaluation Study; Journal Article]. Food Chemistry.

[bb0075] Dent T., LeMinh A., Maleky F. (2024). Comparison of colorimetric methods for measuring the solubility of legume proteins. Gels.

[bb0080] Di Somma A., Moretta A., Canè C., Cirillo A., Duilio A. (2020). Antimicrobial and antibiofilm peptides. Biomolecules.

[bb0085] El Shazely B., Yu G., Johnston P.R., Rolff J. (2020). Resistance evolution against antimicrobial peptides in *Staphylococcus aureus* alters pharmacodynamics beyond the MIC. Frontiers in Microbiology.

[bb0090] Fadimu G.J., Gill H., Farahnaky A., Truong T. (2021). Investigating the impact of ultrasound pretreatment on the physicochemical, structural, and antioxidant properties of lupin protein hydrolysates. Food and Bioprocess Technology.

[bb0095] Franck M., Perreault V., Suwal S., Marciniak A., Bazinet L., Doyen A. (2019). High hydrostatic pressure-assisted enzymatic hydrolysis improved protein digestion of flaxseed protein isolate and generation of peptides with antioxidant activity [Journal Article; Research Support, Non-U.S. Gov't]. Food Research International.

[bb0100] Gharehbeglou P., Sarabandi K., Akbarbaglu Z. (2024). Insights into enzymatic hydrolysis: Exploring effects on antioxidant and functional properties of bioactive peptides from *Chlorella* proteins. Journal of Agriculture and Food Research.

[bb0105] Guan H., Diao X., Jiang F., Han J., Kong B. (2018). The enzymatic hydrolysis of soy protein isolate by Corolase PP under high hydrostatic pressure and its effect on bioactivity and characteristics of hydrolysates [Journal Article]. Food Chemistry.

[bb0110] Hei X., Liu Z., Li S., Wu C., Jiao B., Hu H., Ma X., Zhu J., Adhikari B., Wang Q., Shi A. (2024). Freeze-thaw stability of Pickering emulsion stabilized by modified soy protein particles and its application in plant-based ice cream. International Journal of Biological Macromolecules.

[bb0115] Huan Y., Kong Q., Mou H., Yi H. (2020). Antimicrobial peptides: Classification, design, application and research progress in multiple fields. Frontiers in Microbiology.

[bb0120] Jiang H., Zhang Z., Wang Y., Gao J., Yuan Q., Mao X. (2023). Effects of high hydrostatic pressure treatment on the antigenicity, structural and digestive properties of whey protein. Food Science & Technology.

[bb0125] Johnson Esua O., Sun D., Cheng J., Wang H., Lv M. (2022). Functional and bioactive properties of *Larimichthys polyactis* protein hydrolysates as influenced by plasma functionalized water-ultrasound hybrid treatments and enzyme types. Ultrasonics Sonochemistry.

[bb0130] Kim N. (2017). Production of wheat gluten hydrolyzates by enzymatic process at high pressure. Food Science and Biotechnology.

[bb0135] Li M., Zhao Q., Bu G., Shi S., Zhu T., Chen Y., Zhu J. (2025). Effects of high hydrostatic pressure combined with enzymatic hydrolysis on the allergenicity, structural properties, and antigenic epitopes of soybean glycinin. LWT.

[bb0140] Li T., Lu X., Zhang M., Hu K., Li Z. (2022). Peptide-based nanomaterials: Self-assembly, properties and applications. Bioactive Materials.

[bb0145] Li Z., Xie A., Yan Y., Piao C., Lan A., Wang S., Wu J., Fang X. (2025). Impacts of soy protein isolate hydrolysates on gel properties of high-protein soy yogurt. Food Chemistry.

[bb0150] Liu C., Li M., Zhang W., Chen Y., Chen J., Wu X. (2024). Comparative study of physicochemical and structural properties of plant proteins hydrolyzed by different. Lwt.

[bb0155] Liu F., Li Y., Sun G., Wang C., Liang Y., Zhao X., He J., Mo H. (2022). Influence of ultrasound treatment on the physicochemical and antioxidant properties of mung bean protein hydrolysate. Ultrasonics Sonochemistry.

[bb0160] Liu N., Lin P., Zhang K., Yao X., Li D., Yang L., Zhao M. (2022). Combined effects of limited enzymatic hydrolysis and high hydrostatic pressure on the structural and emulsifying properties of rice proteins. Innovative Food Science &Amp; Emerging Technologies.

[bb0165] Liu S., Hou P., Han Y., Li Y., Wang X., Yang D., Liao X. (2025). Unraveling the molecular mechanisms of high hydrostatic pressure in enhancing emulsifying properties: Insights from main fractionations in pepper seed protein. Food Chemistry.

[bb0170] Liu W., Chen E., Yang L., Peng C., Wang Q., Xu Z., Chen D. (2021). Emerging resistance mechanisms for 4 types of common anti-MRSA antibiotics in Staphylococcus aureus: A comprehensive review. Microbial Pathogenesis.

[bb0175] Liu X., Pan L., Cheng Y., Dong D., Zhao H., Ding X., Yuan C., Cui B. (2025). Effect of high hydrostatic pressure on Alcalase-assisted hydrolysis of soy protein isolate and the anti-aging activity of the hydrolysates. Process Biochemistry.

[bb0180] Lyu S., Chen M., Wang Y., Zhang D., Zhao S., Liu J., Pan F., Zhang T. (2023). Foaming properties of egg white proteins improved by enzymatic hydrolysis: The changes in structure and physicochemical properties. Food Hydrocolloids.

[bb0185] Ma X., Wang Q., Ren K., Xu T., Zhang Z., Xu M., Zhang X. (2024). A review of antimicrobial peptides: Structure, mechanism of action, and molecular optimization strategies. Fermentation-Basel.

[bb0190] Mba I.E., Nweze E.I. (2022). Antimicrobial peptides therapy: An emerging alternative for treating drug-resistant bacteria. The Yale Journal of Biology & Medicine.

[bb0195] Nazir M.A., Mu T.H., Zhang M. (2020). Preparation and identification of angiotensin I-converting enzyme inhibitory peptides from sweet potato protein by enzymatic hydrolysis under high hydrostatic pressure. International Journal of Food Science & Technology.

[bb0200] Ngoh Y., Gan C. (2016). Enzyme-assisted extraction and identification of antioxidative and α-amylase inhibitory peptides from Pinto beans (*Phaseolus vulgaris* cv. Pinto). Food Chemistry.

[bb0205] Osman A., Enan G., Al-Mohammadi A., Abdel-Shafi S., Abdel-Hameid S., Sitohy M.Z., El-Gazzar N. (2021). Antibacterial peptides produced by alcalase from cowpea seed proteins. Antibiotics-Basel.

[bb0210] Pacheco A.F.C., Pacheco F.C., Nalon G.A., Cunha J.S., Andressa I., Paiva P.H.C., de Castro Leite Júnior B.R. (2025). Impact of ultrasonic pretreatment on pumpkin seed protein: Effect on protease activities, protein structure, hydrolysis kinetics and functional properties. Food Research International.

[bb0215] Panda R., Tetteh A.O., Pramod S.N., Goodman R.E. (2015). Enzymatic hydrolysis does not reduce the biological reactivity of soybean proteins for all allergic subjects. Journal of Agricultural and Food Chemistry.

[bb0220] Piewngam P., Khongthong S., Roekngam N., Theapparat Y., Sunpaweravong S., Faroongsarng D., Otto M. (2023). Probiotic for pathogen-specific *Staphylococcus aureus* decolonisation in Thailand: A phase 2, double-blind, randomised, placebo-controlled trial. The Lancet. Microbe.

[bb0225] Queirós R.P., Saraiva J.A., Da Silva J.A.L. (2018). Tailoring structure and technological properties of plant proteins using high hydrostatic pressure. Critical Reviews in Food Science and Nutrition.

[bb0230] Rao T.J., Mounika A., Shanmugam A. (2024). Improved bioactivity and functional qualities of millet protein enzyme hydrolysates by ultrasonication. Industrial & Engineering Chemistry Research.

[bb0235] Rivero-Pino F., Leon M.J., Millan-Linares M.C., Montserrat-de la Paz S. (2023). Antimicrobial plant-derived peptides obtained by enzymatic hydrolysis and fermentation as components to improve current food systems. Trends in Food Science & Technology.

[bb0240] Rodriguez A., Chen B., Pangloli P., D’Souza D., Krishnan H.B., Dia V.P. (2025). Enzymatic hydrolysis altered the physicochemical and immunogenic profile of protein-based ingredients derived from industrial hempseed (*Cannabis sativa* L.). Food Bioscience.

[bb0245] Saraiva M.A. (2020). Interpretation of α-synuclein UV absorption spectra in the peptide bond and the aromatic regions. Journal of Photochemistry and Photobiology. B, Biology.

[bb0250] Shao F., Zhang Y., Wan X., Duan Y., Cai M., Hu K., Zhang H. (2024). Regulation in protein hydrophobicity via whey protein-zein self-assembly for improving the techno-functional properties of protein. Food Chemistry.

[bb0255] Shuai X., Gao L., Geng Q., Li T., He X., Chen J., Dai T. (2022). Effects of moderate enzymatic hydrolysis on structure and functional properties of pea protein. Foods.

[bb0260] Sirelkhatim A., Mahmud S., Seeni A., Kaus N.H.M., Ann L.C., Bakhori S.K.M., Mohamad D. (2015). Review on zinc oxide nanoparticles: Antibacterial activity and toxicity mechanism. Nano-Micro Letters.

[bb0265] Sneideris T., Erkamp N.A., Ausserwöger H., Saar K.L., Welsh T.J., Qian D., Knowles T.P.J. (2023). Targeting nucleic acid phase transitions as a mechanism of action for antimicrobial peptides. Nature Communications.

[bb0270] Sun S. (2024). Progress in the identification and design of novel antimicrobial peptides against pathogenic microorganisms. Probiotics and Antimicrobial Proteins.

[bb0275] Sun Y., Chang R., Li Q., Li B. (2016). Isolation and characterization of an antibacterial peptide from protein hydrolysates of Spirulina platensis. European Food Research and Technology.

[bb0280] Tacias-Pascacio V.G., Morellon-Sterling R., Siar E., Tavano O., Berenguer-Murcia Á., Fernandez-Lafuente R. (2020). Use of Alcalase in the production of bioactive peptides: A review. International Journal of Biological Macromolecules.

[bb0285] Tan P., Fu H., Ma X. (2021). Design, optimization, and nanotechnology of antimicrobial peptides: From exploration to applications. Nano Today.

[bb0290] Tincho M.B., Morris T., Meyer M., Pretorius A. (2020). Antibacterial activity of rationally designed antimicrobial peptides. International Journal of Microbiology.

[bb0295] Tong X., Cao J., Tian T., Lyu B., Miao L., Lian Z., Cui W., Liu S., Wang H., Jiang L. (2022). Changes in structure, rheological property and antioxidant activity of soy protein isolate fibrils by ultrasound pretreatment and EGCG. Food Hydrocolloids.

[bb0300] Tong Z., Zhang L., Ling J., Jian Y., Huang L., Deng D. (2014). An in vitro study on the effect of free amino acids alone or in combination with nisin on biofilms as well as on planktonic bacteria of *Streptococcus mutans*. PLoS One.

[bb0305] Wang F., Ma Y., Wang Y., Zhao L., Liao X. (2021). Physicochemical properties of seed protein isolates extracted from pepper meal by pressure-assisted and conventional solvent defatting. Food & Function.

[bb0310] Wang J., Su Y., Jia F., Jin H. (2013). Characterization of casein hydrolysates derived from enzymatic hydrolysis. BMC Chemistry.

[bb0315] Wang J., Yang T., Yu W., Zhou J., Bian Y., Li S., Chen Y. (2025). Pea power against microbes: Elucidating the characteristics and antimicrobial mechanisms of antimicrobial peptides obtained through enzymatic hydrolysis of pea protein isolate. Food Bioscience.

[bb0320] Wang S., Wang T., Sun Y., Cui Y., Yu G., Jiang L. (2021). Effects of high hydrostatic pressure pretreatment on the functional and structural properties of Rice bran protein hydrolysates. Foods.

[bb0325] Wang W., Yang P., Rao L., Zhao L., Wu X., Wang Y., Liao X. (2022). Effect of high hydrostatic pressure processing on the structure, functionality, and nutritional properties of food proteins: A review. Comprehensive Reviews in Food Science and Food Safety.

[bb0330] Xu Y., Galanopoulos M., Sismour E., Ren S., Mersha Z., Lynch P., Almutaimi A. (2020). Effect of enzymatic hydrolysis using endo- and exo-proteases on secondary structure, functional, and antioxidant properties of chickpea protein hydrolysates. Journal of Food Measurement & Characterization.

[bb0335] Yang J., Zhu B., Dou J., Ning Y., Wang H., Huang Y., Li Y., Qi B., Jiang L. (2023). pH and ultrasound driven structure-function relationships of soy protein hydrolysate. Innovative Food Science &Amp; Emerging Technologies.

[bb0340] Yang L., Zhu W., Ding Y., Zhuang Y. (2025). New insights into altering the structures and improving the functional properties of walnut protein: Synergistic high-pressure homogenization/limited enzymatic hydrolysis. Innovative Food Science &Amp; Emerging Technologies.

[bb0345] Yin S., Tang C., Wen Q., Yang X. (2009). Effects of acylation on the functional properties and in vitro trypsin digestibility of red kidney bean (*Phaseolus vulgaris* L.) protein isolate. Journal of Food Science.

[bb0350] Yolandani, Ma H., Li Y., Liu D., Zhou H., Liu X., Wan Y., Zhao X. (2023). Ultrasound-assisted limited enzymatic hydrolysis of high concentrated soy protein isolate: Alterations on the functional properties and its relation with hydrophobicity and molecular weight [Journal Article]. Ultrasonics Sonochemistry.

[bb0355] Zhang L., Song C., Chang J., Wang Z., Meng X. (2022). Optimization of protein hydrolysates production from defatted peanut meal based on physicochemical characteristics and sensory analysis. LWT.

[bb0360] Zhang M., Mu T. (2017). Identification and characterization of antioxidant peptides from sweet potato protein hydrolysates by Alcalase under high hydrostatic pressure. Innovative Food Science & Emerging Technologies.

[bb0365] Zhang Q., Yan Z., Meng Y., Hong X., Shao G., Ma J., Fu C. (2021). Antimicrobial peptides: Mechanism of action, activity and clinical potential. Military Medical Research.

[bb0370] Zhang R., Xu L., Dong C. (2022). Antimicrobial peptides: An overview of their structure, function and mechanism of action. Protein and Peptide Letters.

[bb0375] Zhang X., Wang L., Chen Z., Li Y., Luo X., Li Y. (2020). Effect of high energy electron beam on proteolysis and antioxidant activity of rice proteins. Food & Function.

[bb0380] Zhang Y., Jing X., Chen Z., Wang X. (2022). Purification and identification of antioxidant peptides from millet gliadin treated with high hydrostatic pressure. Food Science & Technology.

[bb0385] Zhang Y., Kong Y., Xu W., Yang Z., Bao Y. (2023). Electron beam irradiation alters the physicochemical properties of chickpea proteins and the peptidomic profile of its digest. Molecules.

[bb0390] Zhang Z., Shen Y., Xin G., Deng W., Tan H., Adel Ashour A., Li D. (2024). The effect of static magnetic field on inducing the binding of bovine serum albumin and cyanidin-3-O-glucoside. Food Innovation and Advances.

[bb0395] Zhao F., Liu X., Ding X., Dong H., Wang W. (2019). Effects of high-intensity ultrasound pretreatment on structure, properties, and Enzymolysis of soy protein isolate. Molecules (Basel, Switzerland).

[bb0400] Zhao Q., He L., Wang X., Ding X., Li L., Tian Y., Huang A. (2022). Characterization of a novel antimicrobial peptide isolated from *Moringa oleifera* seed protein hydrolysates and its membrane damaging effects on *Staphylococcus aureus*. Journal of Agricultural and Food Chemistry.

[bb0405] Zhao Q., Shi Y., Wang X., Huang A. (2020). Characterization of a novel antimicrobial peptide from buffalo casein hydrolysate based on live bacteria adsorption. Journal of Dairy Science.

[bb0410] Zheng Y., Wang D., Zhou Y., Yuen M., Yuen T., Yuen H., Peng Q. (2024). Characterization and angiotensin-converting enzyme inhibitory activity of peptides of seabuckthorn (*Hippophae rhamnoides* L.) seed meal. Food Innovation and Advances.

